# Testing the Ability of Non-Methylamine Osmolytes Present in Kidney Cells to Counteract the Deleterious Effects of Urea on Structure, Stability and Function of Proteins

**DOI:** 10.1371/journal.pone.0072533

**Published:** 2013-09-09

**Authors:** Sheeza Khan, Zehra Bano, Laishram R. Singh, Md. Imtaiyaz Hassan, Asimul Islam, Faizan Ahmad

**Affiliations:** 1 Centre for Interdisciplinary Research in Basic Sciences, Jamia Millia Islamia, New Delhi, India; 2 Dr. B. R. Ambedkar Center for Biomedical Research, University of Delhi, Delhi, India; Russian Academy of Sciences, Institute for Biological Instrumentation, Russian Federation

## Abstract

Human kidney cells are under constant urea stress due to its urine concentrating mechanism. It is believed that the deleterious effect of urea is counteracted by methylamine osmolytes (glycine betaine and glycerophosphocholine) present in kidney cells. A question arises: Do the stabilizing osmolytes, non-methylamines (myo-inositol, sorbitol and taurine) present in the kidney cells also counteract the deleterious effects of urea? To answer this question, we have measured structure, thermodynamic stability (Δ*G*_D_^o^) and functional activity parameters (*K*_m_ and *k*_cat_) of different model proteins in the presence of various concentrations of urea and each non-methylamine osmolyte alone and in combination. We observed that (i) for each protein myo-inositol provides perfect counteraction at 1∶2 ([myo-inositol]:[urea]) ratio, (ii) any concentration of sorbitol fails to refold urea denatured proteins if it is six times less than that of urea, and (iii) taurine regulates perfect counteraction in a protein specific manner; 1.5∶2.0, 1.2∶2.0 and 1.0∶2.0 ([taurine]:[urea]) ratios for RNase-A, lysozyme and α-lactalbumin, respectively.

## Introduction

The waste product, urea plays a key role in the osmoregulation of reno-medullary cells in diuretic and antidiuretic conditions. As part of the urinary concentrating mechanism, renal inner medullary cells are normally exposed to variable and often very high interstitial levels of urea, yet they survive and function. The urea concentration under diuretic condition is 400–600 mM in the mammalian kidneys including human [1] and reaches up to 3–4 M under antidiuretic conditions [2]. High urea is harmful to cells, altering many enzymatic functions or even killing them by apoptosis [3], [4]. Urea is a chaotropic agent that disrupts non-covalent interactions responsible for the globular structure of proteins [5], [6], [7], [8] and also influences enzyme kinetic properties such as maximal velocity (*V*_max_) and *K*_m_ [6], [7]. In addition to the chaotropic nature, high urea concentration can also bring about post-translational modification of large number of proteins either by carbamoylation or carbonylation near physiological pH and temperature [9], [10].

It is generally believed that the deleterious effect of urea on macromolecules (in kidney cells) is offset by the accumulation of small organic compounds in the osmoticum (called osmolytes). In fact, the principal osmolytic compounds present in the urea-rich kidney cells consists of methylamines (glycerophosphocholine (GPC) and glycine betaine (GB)) and non-methylamines (myo-inositol, sorbitol and taurine). The physiological concentration of each non-methylamine is different in cells of different species [11]–[22]. In mammalian kidney cells concentrations of myo-inositol, sorbitol and taurine are 10–21, 5–16 and less than 10–20 mosmol/L, respectively. It has been observed that concentration of non-methylamines does not change during urea stress [23], [24]. On the contrary, methylamine concentration increases with an increase in urea concentration [25]. The effect of methylamine osmolytes in offsetting the effects of urea on proteins (in terms of thermodynamic stability, structure and activity) has earlier been investigated. It is known that when the concentration ratio of methylamines to urea is 1∶2, their opposing effects are independently additive, preserving macromolecular structure and function [7], [26].

Earlier studies have shown that concentrations of non-methylamines in kidney cells increases with an increase in NaCl concentration, but it remains unchanged with an increase in urea concentration [23], [24]. A question arises: Do non-methylamines have the ability to offset the deleterious effect of urea on protein structure and function? In this communication we have investigated the counteractive ability of all the non-methylamine osmolytes present in the osmoticum of the reno-medullary cells in terms of structure, stability and function of three proteins namely, RNase-A, lysozyme and α-lactalbumin (α-LA). To our surprise we discovered that the non-methylamine osmolytes, myo-inositol and taurine have the counteracting ability on the three proteins investigated. Myo-inositol brings about perfect counteraction at 1∶2 ratio (myo-inositol: urea) on all the proteins; while taurine shows protein-specific counteraction ratios. This study shows that in addition to methylamines, myo-inositol and taurine might be counteracting osmolytes (osmolytes that can reverse the denaturing effect of urea on macromolecules) *in vivo*.

## Methods

### Reagents

Commercial lyophilized preparations of ribonuclease-A (type III-A) from bovine pancreas (RNase-A), chicken egg-white lysozyme and α-lactalbumin (type I) from bovine milk were purchased from Sigma Chemical Co. Urea was procured from ICN Biomedicals Inc. Sorbitol, myo-inositol, and taurine were also obtained from Sigma Chemical Co. Guanidinium chloride (GdmCl) was ultrapure sample from Schwarz/Mann. These and other chemicals, which were of analytical grade, were used without further purification.

Stock solution of each protein was dialyzed extensively against 0.1 M KCl pH 7.0 at 4°C. Apo-α-lactalbumin (α-LA) was prepared by adding 4.0 mM EGTA to the dialyzed solution of holoprotein (with Ca^2+^ bound). Protein stock solutions were filtered using 0.22 µm Millipore filter paper. All three proteins gave single band during polyacrylamide gel electrophoresis. Protein concentration was determined experimentally using molar absorption coefficient, *ε* (M^−1^cm^−1^) value of 9800 at 277.5 nm for RNase-A [27], 39000 at 280 nm for lysozyme [28] and 29210 at 280 nm for α-LA [29]. Urea stock solution was prepared fresh for each day. Concentrations of the stock solutions of urea and GdmCl were determined by refractive index measurements [30]. All solutions for optical measurements were prepared in degassed 50 mM buffers containing 0.1 M KCl. For various pH ranges, the buffers used were KCl-HCl buffer (pH 2.0), citrate buffer (pH 3.0), acetate buffer (pH 4.0) and cacodylic acid buffer (pH range 5.0–7.0). Since, the change in pH may also occur on heating, or on addition of urea, the pH of the solution was, therefore, measured after the denaturation experiment; the variation in the pH was found to be insignificant.

### Thermal Denaturation Studies

Thermal denaturation studies were carried out in Jasco V-560 UV/Vis spectrophotometer equipped with a peltier type temperature controller (ETC-505T), with a heating rate of 1°C/min. This scan rate was found to provide adequate time for equilibration. Each sample was heated from 20 to 85°C. Change in the absorbance with increasing temperature was followed at 287 nm for RNase-A, 300 nm for lysozyme and 295 nm for α-LA. About 650 data points of each transition curve were collected. After denaturation, the sample was immediately cooled down to measure reversibility. All solution blanks showed negligible change in absorbance with temperature and were, therefore, neglected during the data analysis. The raw absorbance data were converted into Δ*ε*_λ_, the difference molar absorption coefficient (M^−1^ cm^−1^) at a given wavelength, λ (*ε*_λ_ at any temperature *T* – *ε*_λ_ at 20°C). Each heat-induced transition curve (plot of Δ*ε* versus temperature) was analyzed for *T*_m_ (the midpoint of heat denaturation) and Δ*H*_m_ (the enthalpy change of denaturation at *T*_m_) using a non-linear least-squares analysis according to the relation [31],
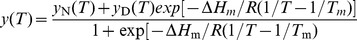
(1)where *y*(*T*) is the optical property at temperature *T* (Kelvin); *y*_N_(*T*) and *y*_D_(*T*) are the optical properties of the native and denatured protein molecules, respectively; and *R* is the gas constant. In the analysis of the transition curve, it was assumed that a parabolic function describes the dependence of the optical properties of the native and denatured protein molecules (i.e., *y*_N_(*T*) = *a*_N_+*b*_N_ *T*+*c*_N_ *T* ^2^, and *y*_D_(*T*) = *a*_D_+*b*_D_ *T*+*c*_D_ *T* ^2^, where *a*_N_, *b*_N_, *c*_N_, *a*_D_, *b*_D_, and *c*_D_ are temperature-independent coefficients). The reason for using cubic dependencies of baselines is an observation that there exists and excellent agreement between a thermodynamic parameters obtained from such an analysis of the thermal transition curve and from DSC measurements [31], [32]. Δ*G*_D_ (*T*), the value of Δ*G*_D_ at any temperature *T* was estimated using the known values of *T*_m_, Δ*H*_m_ and Δ*C*_p_ (constant-pressure heat capacity change on denaturation) in the Gibbs-Helmholtz equation, 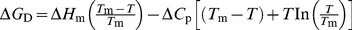
(2)


### CD Measurements

The far- and near-UV circular dichroism (CD) spectra of RNase A, lysozyme, and α-LA in the presence and absence of the co-solvents were measured in J-715 (Jasco spectropolarimeter) equipped with a Peltier-type temperature controller (PTC-348-WI) at least three times at 25 and 85°C. At least six scans were accumulated to average out a spectrum to improve upon the signal-to-noise ratio in each case, including the base line. Each spectrum of a protein was corrected for the contribution of its blank in the entire wavelength range. The CD signal at each wavelength was converted into mean residue ellipticity (deg cm^2^ dmol^–1^) using the relation,
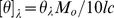
(3)where *θ*_λ_ is the observed ellipticity (millidegrees) at the wavelength λ, *M*_o_ is the mean residue weight of the protein, *c* is the protein concentration (mg/cm^3^), and *l* is the path length (centimeters). The protein concentration for the CD measurements was in the range 0.2–0.3 mg/cm^3^. A 0.1 cm path length cell was used for the far-UV CD measurements, and a 1.0 cm path length cell was used for the near-UV CD measurements. It should be noted that the CD instrument was routinely calibrated with D-10-camphorsulfonic acid.

### Measurements of Enzymatic Activity Parameters

The effect of different concentrations of urea and each stabilizing osmolyte alone, and different urea-osmolyte mixtures on the kinetic parameters (*K*_m_ and *k*_cat_) for the lytic activity of lysozyme towards *M. luteus* cell walls was measured at pH 7.0 and 25°C by the method of Maurel & Douzou [33]. The substrate and the enzyme were pre-incubated in a given concentration of urea, each stabilizing osmolyte (myo-inositol, sorbitol and taurine) alone and in combined osmolyte-urea mixtures. The decrease in absorbance of a turbid aqueous suspension of *M. luteus* on the addition of lysozyme was recorded at 450 nm in Jasco V-660 UV/Visible spectrophotometer with constant stirring. The rate of lysis was deduced from the slope of the linear part of the recordings, usually over the first 30 sec, wherein 10 to 20% of the substrate was lysed. At 450 nm the apparent specific absorbance of an aqueous suspension of *M. luteus* cell walls was *ε*_450_ = 0.65×10^−2^ mg l^−1^. The rate of lysis was defined as the weight of cells lysed (in mg ml^−1^) per sec and per mol of lysozyme. The assay media were directly prepared in 1 cm path length glass cell. In a typical experiment, different concentrations, ranging from 10 to 200 µl, of a stock aqueous suspension of *M. luteus* cells (3 mg m1^−1^), were added, and the final volume was adjusted to 3 ml with 50 mM cacodylate buffer, pH 7.0. The cell was then placed into the spectrophotometer cell holder maintained at 25±0.1°C. The reaction was initiated by the addition of 20 µl of a lysozyme stock solution (1 mg/ml, in 50 mM cacodylate buffer, pH 7.0). The kinetic parameters *K*_m_ (µg m1^−1^) and *V*_max_ (s^−1^ M^−1^) were determined from Michaelis-Menten plots using equation,
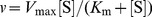
(4)where *ν* is the initial velocity, and [S] is the substrate concentration. In these experiments *M. luteus* concentration was varied from 10 to 200 µg m1^−1^ and the lysozyme concentration was kept constant at 0.45 µM. The value of *k*_cat_ (mg ml^−1^ s^−1^ M^−1^) is the product of *V*_max_ and enzyme concentration.

RNase-A activity was assayed to see the effect of different concentrations of urea and each osmolyte alone, and different concentrations of each osmolyte-urea mixture on its kinetic parameters (*K*_m_ and *k*_cat_) as described previously [34]. For this, the substrate (2′–3′ cyclic monophosphate) and enzyme solutions were pre-incubated in a given concentration of urea and each osmolyte alone and in each osmolyte-urea mixture. RNase-A mediated hydrolysis of the substrate in the concentration range 0.05–0.50 mg ml^−1^ was followed by measuring the change in absorbance at 292 nm at 25.0±0.1°C for 20 min in Jasco V-660 UV/Vis spectrophotometer. From each progress curve at a given substrate concentration, initial velocity (ν) was determined from the linear portion of the progress curve, usually first 30 sec. The plot of ν versus [S] (in mM) at each co-solvent concentration was analyzed for *K*_m_ and *V*_max_ using equation (4). From this analysis the values of *k*_cat_ were determined.

## Results

**Figure 1:**
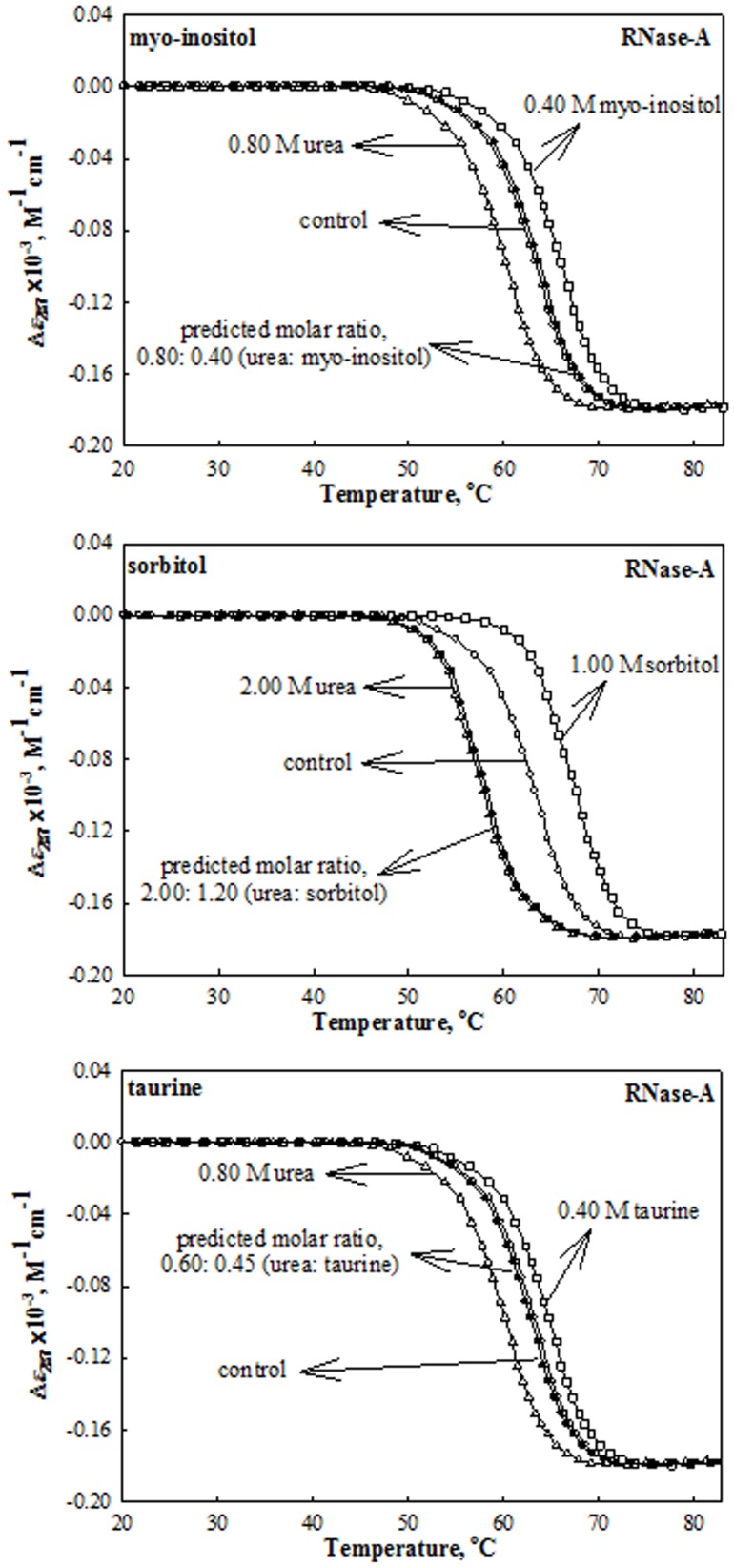
Effect of urea, stabilizing osmolytes and their mixtures (at predicted ratio for perfect counteraction) on RNase-A at pH 7.0 and 25°C. Representative thermal denaturation curves of RNase-A in the presence of the indicated concentrations of urea, osmolytes and their mixtures at predicted ratios given in Table 2. To maintain clarity all data points and all transition curves are not shown.

**Figure 2:**
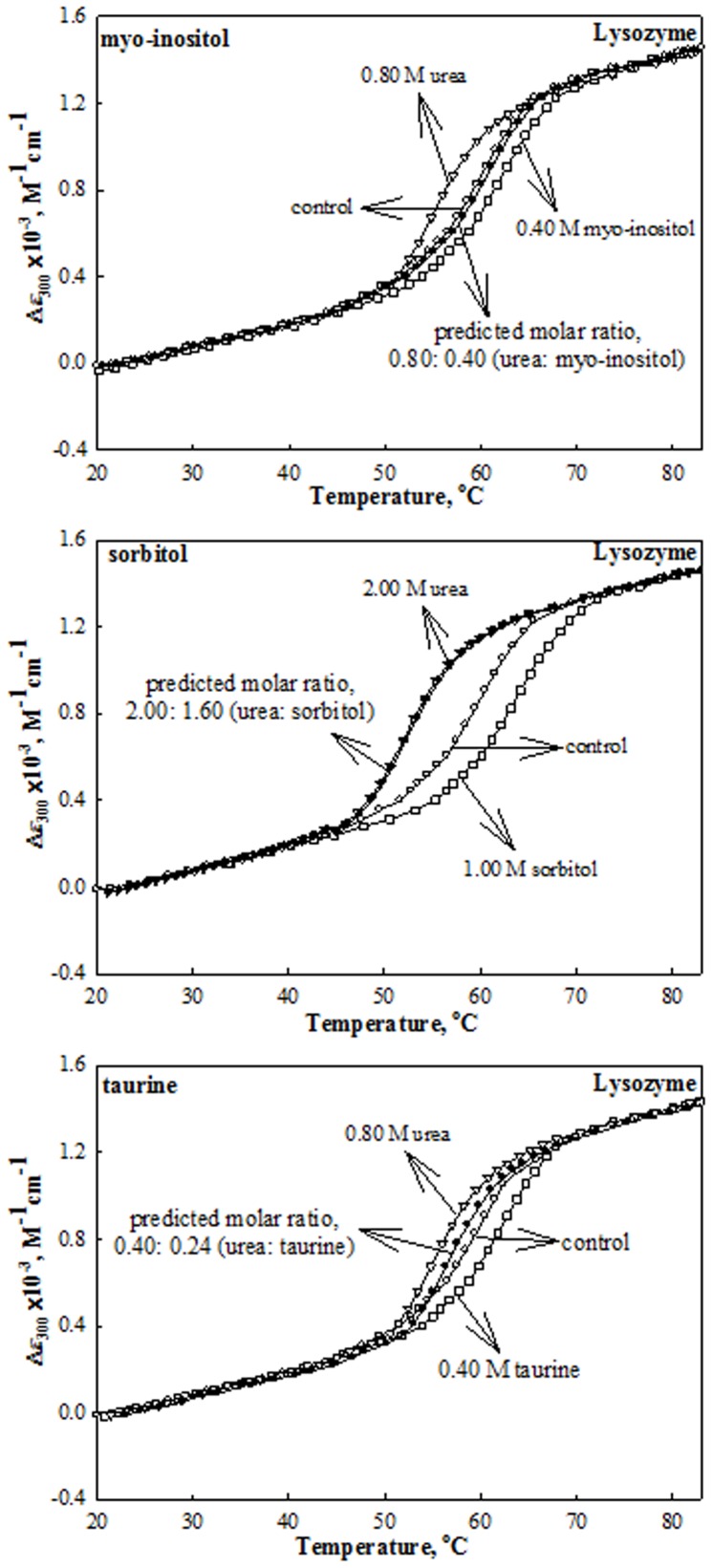
Effect of urea, stabilizing osmolytes and their mixtures (at predicted ratio for perfect counteraction) on Lysozyme at pH 7.0 and 25°C. Representative thermal denaturation curves of Lysozyme in the presence of the indicated concentrations of urea, osmolytes and their mixtures at predicted ratios given in Table 2. To maintain clarity all data point and all transition curves are not shown.

**Figure 3:**
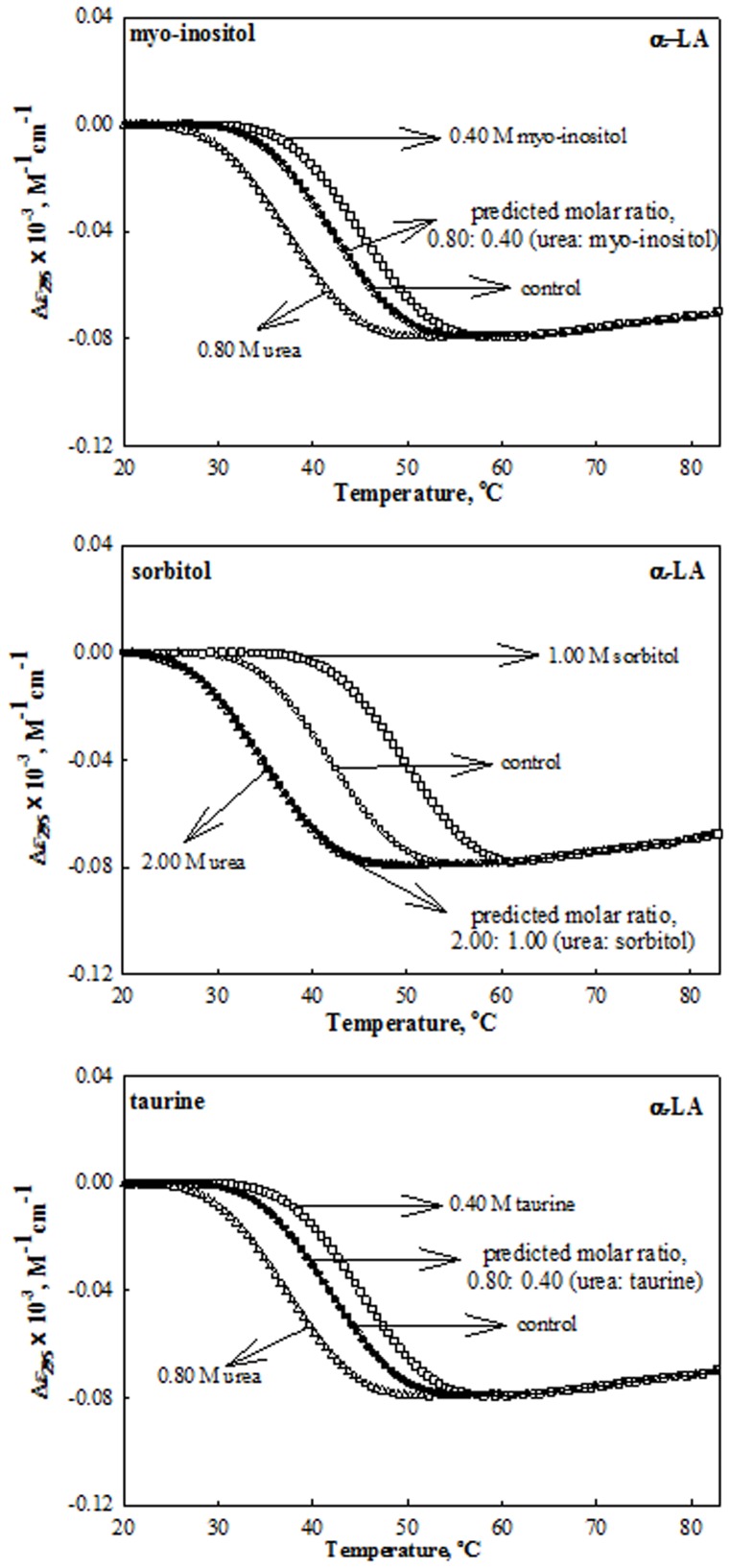
Effect of urea, stabilizing osmolytes and their mixtures (at predicted ratio for perfect counteraction) on α-LA at pH 7.0 and 25°C. Representative thermal denaturation curves of α-LA in the presence of the indicated concentrations of urea, osmolytes and their mixtures at predicted ratios given in Table 2. To maintain clarity all data points and all transition curves are not shown.

The role of non-methylamine osmolytes (myo-inositol, sorbitol and taurine) in counteracting the effects of urea on protein stability was investigated by measuring the heat-induced denaturation curves of the proteins (RNase-A, lysozyme and α-LA) at different pH values in the presence of each of the osmolytes and urea at various concentrations (0.1–1.0 M for stabilizing osmolytes and 0.2–2.0 M in case of urea). We have intentionally chosen these urea concentrations as all the three proteins exist in native state in the presence of these urea concentrations. To investigate the effect of these co-solutes on the protein stability, heat-induced denaturation curves were monitored by following changes in Δ*ε_λ_*, the difference in molar absorption co-efficient at the wavelength, *λ*; Δ*ε*_287_ for RNase-A, Δ*ε*_300_ for lysozyme and Δ*ε*_295_ for α-LA. Figures 1 to 3 show the representative heat-induced denaturation curves of RNase-A, lysozyme and α-LA in the absence and presence of the highest concentration of these co-solutes at pH 7.0. It should be noted that we could not measure the effects of myo-inositol and taurine beyond 0.4 M due to their limited solubility. Denaturation of each protein was reversible in the presence and absence of urea and each stabilizing osmolyte making it amenable for the thermodynamic analysis. All heat-induced denaturation curves were analyzed for *T*_m_ and Δ*H*_m_ (enthalpy change at *T*_m_) using equation (1). This analysis involves fitting the entire (Δ*ε*, *T*) data of a transition curve to equation (1) with all eight free parameters (*a*_N_, *b*_N_, *c*_N_, *a*_D_, *b*_D_, *c*_D_, Δ*H*_m_ and *T*_m_). Table 1 shows values of Δ*H*_m_ and *T*_m_ of each protein at the given co-solute concentrations. In the case of lysozyme we could not get a complete transition curve in the measurable temperature range in the presence and absence of osmolytes at pH 7.0. To bring down the thermal denaturation curves in the measurable temperature range, measurements were carried out in the presence of 2.0 M GdmCl. The contribution of the 2.0 M GdmCl to the observed thermodynamic parameters was corrected using the method published earlier [35]. Therefore, the values of *T*_m_ and Δ*H*_m_ shown in Table 1 (at pH 7.0) are the corrected values for the contribution of GdmCl.

**Table 1:** Thermodynamic parameters associated with the thermal unfolding of RNase-A, Lysozyme and α-LA at pH 7.0.a, b, c.

	RNase-A			Lysozyme			α-LA		
[Osmolyte]	Δ*G*_D_^o^	*T*_m_	Δ*H*_m_	Δ*G*_D_^o^	*T*_m_	Δ*H*_m_	Δ*G*_D_^o^	*T*_m_	Δ*H*_m_
M	kcal mol^−1^	°C	kcal mol^−1^	kcal mol^−1^	°C	kcal mol^−1^	kcal mol^−1^	°C	kcal mol^−1^
*Urea*									
0.00	10.41	63.0	116	13.00	86.0	129	2.01	42.3	50
0.50	9.93	61.6	114	12.67	84.4	127	1.54	40.1	44
1.00	9.28	60.0	111	12.31	83.2	125	1.47	38.0	42
1.50	8.72	57.9	109	12.06	82.4	123	1.21	36.2	39
2.00	8.34	56.3	108	11.69	80.9	121	0.91	35.0	35
		(56.0)	(109)		(80.7)	(121)		(34.8)	(34)
*Myo-inositol*									
0.10	10.67	63.7	117	13.17	86.4	129	2.12	43.2	51
0.20	10.93	64.3	118	13.31	86.9	130	2.21	43.8	52
0.30	11.05	65.0	118	13.43	87.3	130	2.36	44.6	53
0.40	11.29	65.7	119	13.56	87.8	131	2.43	45.3	54
		(66.0)	(118)		(88.0)	(130)		(45.5)	(56)
*Urea+myo-inositol*									
0.20+0.10	10.36	63.1	115	13.00	86.1	129	2.00	42.1	50
0.40+0.20	10.37	63.3	115	12.95	86.6	128	1.93	42.4	49
0.60+0.30	10.33	63.5	114	13.06	86.5	128	2.02	42.2	50
0.80+0.40	10.36	63.8	114	12.84	86.7	127	1.90	42.1	48
		(63.7)	(114)		(86.6)	(126)		(42.1)	(50)
*Sorbitol*									
0.25	10.93	64.3	118	13.30	86.7	131	2.24	44.1	52
0.50	11.36	65.4	120	13.49	87.6	131	2.55	45.9	55
0.75	11.73	66.9	121	13.80	88.5	132	2.79	47.7	57
1.00	12.27	68.5	123	13.83	89.7	132	3.07	50.1	59
		(68.1)	(122)		(89.9)	(131)		(50.3)	(58)
*Urea+sorbitol* c	9.80	61.5	113	12.79	84.4	128	1.67	40.3	45
0.50+ v	9.46	60.1	112	12.26	83.2	125	1.43	38.2	42
1.00+ w	8.87	57.9	110	11.74	82.0	122	1.21	36.2	39
1.50+ x	8.22	56.2	107	11.53	80.5	121	0.92	35.1	36
2.00+ y		(55.8)	(107)		(80.3)	(121)		(34.8)	(35)
*Taurine*									
0.10	10.48	63.4	116	13.02	86.8	129	2.09	42.8	51
0.20	10.64	63.7	117	13.14	87.3	130	2.19	44.0	51
0.30	10.83	64.1	118	13.15	87.8	130	2.29	44.4	52
0.40	10.94	64.5	118	13.33	88.3	131	2.40	45.4	53
		(64.0)	(120)		(88.5)	(131)		(45.1)	(53)
*Urea+taurine* c									
0.20+ v	10.30	62.8	116	13.00	85.8	129	2.05	42.1	51
0.40+ w	10.11	62.4	115	12.82	85.7	128	1.91	42.4	48
0.60+ x	10.11	62.4	115		(85.5)	(129)	2.01	42.2	50
		(62.5)	(114)				1.94	42.1	49
								(42.0)	(50)

a Errors in Δ*G*_D_^o^, Δ*H*_m_ and *T*_m_ from triplicate measurements are 5–9%, 2–5% and 0.1–0.6%, respectively.

b Values in the parentheses are from [*θ*]_222_ measurements.

c v, w, x and y represent concentrations of the osmolyte predicted from results given in Table 2 (see text). For instance, values of y for sorbitol are 1.20, 1.60 and 1.00 for RNase-A, lysozyme and α-LA, respectively.

**Figure 4:**
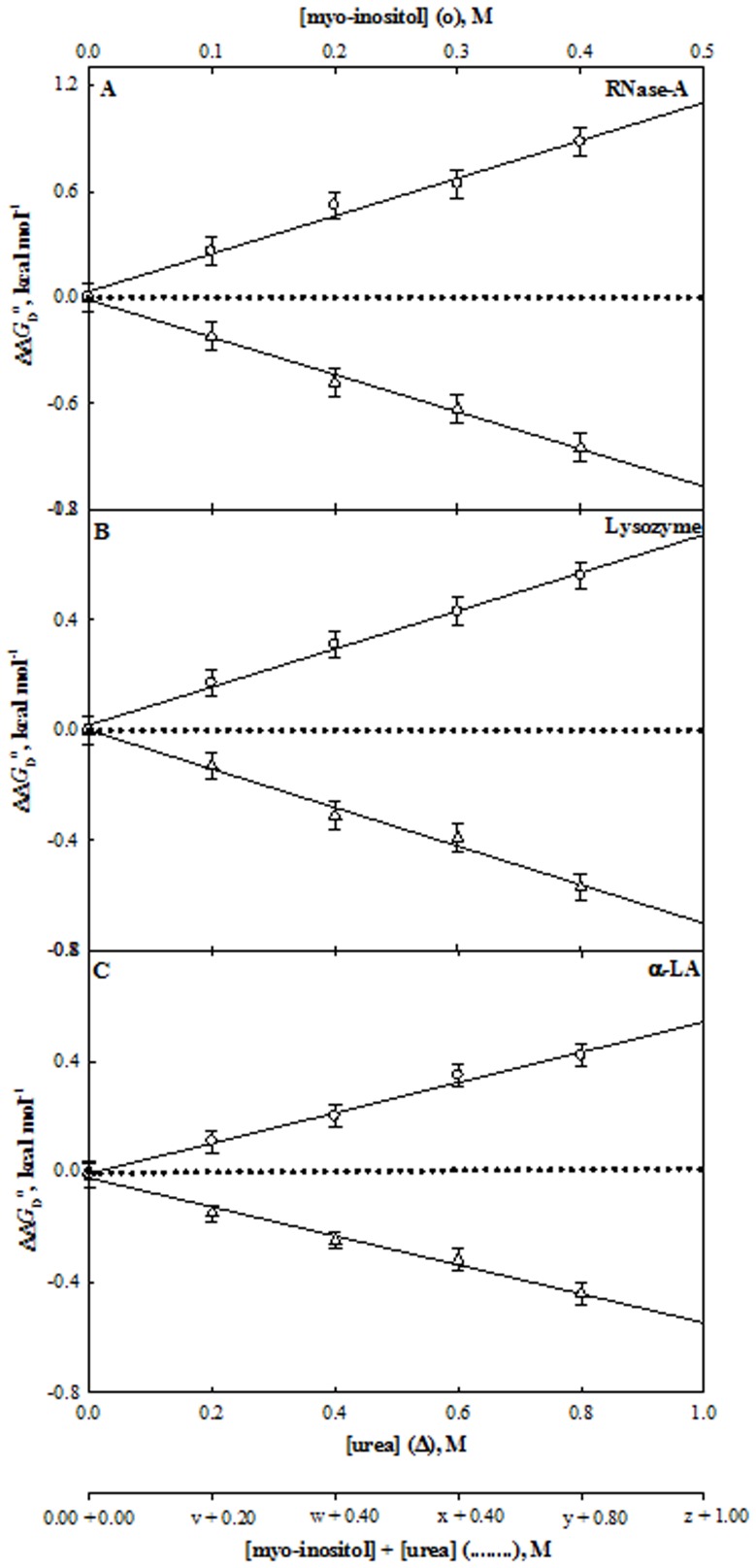
Effect of myo-inositol, urea and their mixtures on stability of proteins at pH 7.0 and 25°C. Plots of ΔΔ*G*_D_^o^ versus [myo-inositol] (○) and [urea] (Δ). The dotted line represents the ΔΔ*G*_D_^o^ values measured at the predicted ratios (see Table 2). The values of v, w, x, y and z of each protein are those predicted from results given in Table 2. For instance, value of y for myo-inositol is 0.40 M for RNase-A, lysozyme and α-LA.

**Figure 5:**
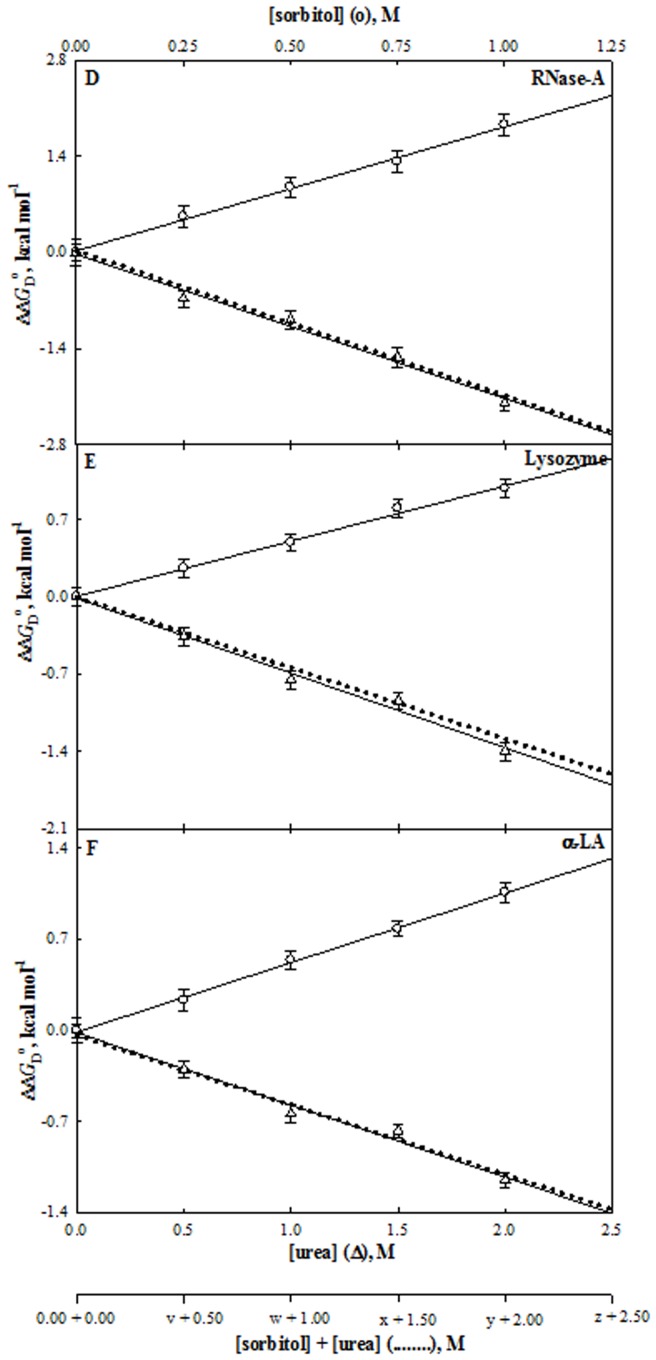
Effect of sorbitol, urea and their mixtures on stability of proteins at pH°C. Plots of ΔΔ*G*_D_^o^ versus [sorbitol] (○) and [urea] (Δ). The dotted line represents the ΔΔ*G*_D_^o^ values measured at the predicted ratios (see Table 2). The values of v, w, x, y and z of each protein are those predicted from results given in Table 2. For instance, values of y for sorbitol are 1.20, 1.60 and 1.00 M for RNase-A, lysozyme and α-LA, respectively.

**Figure 6:**
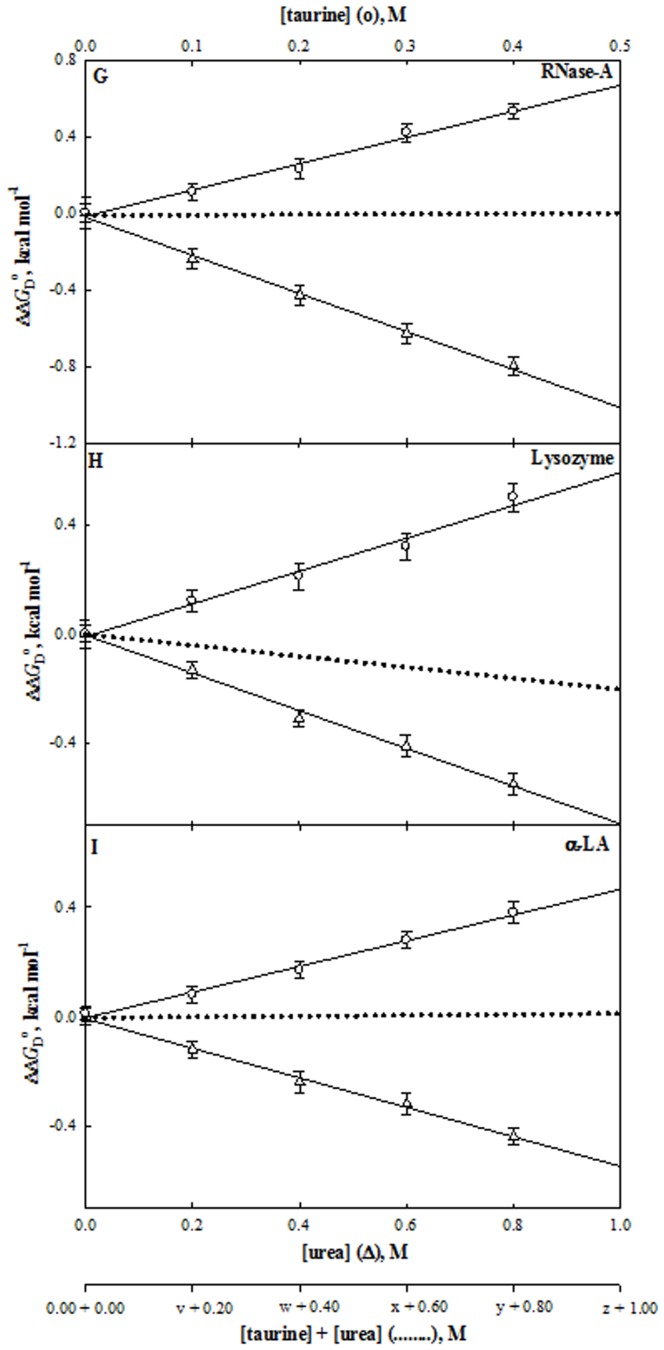
Effect of taurine, urea and their mixtures on stability of proteins at pH°C. Plots of ΔΔ*G*_D_^o^ versus [taurine] (○) and [urea] (Δ). The dotted line represents the ΔΔ*G*_D_^o^ values measured at the predicted ratios (see Table 2). The values of v, w, x, y and z of each protein are those predicted from results given in Table 2. For instance, values of y for taurine are 0.60, 0.48 and 0.40 M for RNase-A, lysozyme and α-LA, respectively.

Values of Δ*C*_p_ (constant-pressure heat capacity change) of the protein were determined by plotting Δ*H*_m_ and *T*_m_ values in the absence and presence of each co-solute, generated at five different pH values (see Tables S1–S12 in Appendix S1), for Δ*C*_p_ is independent of pH [36]. Values of Δ*G*_D_^o^, (value of Δ*G*_D_ at 25°C) estimated at pH 7.0 using equation (2), are given in Table 1. Figures 4 to 6 show representative plots of ΔΔ*G*_D_^o^ (Δ*G*_D_^o^ in the presence of co-solute - Δ*G*_D_^o^ in the absence of co-solute) versus [co-solute], the molar concentration of the co-solute. It is seen in these figures that ΔΔ*G*_D_^o^ of the proteins increases in the presence of the stabilizing osmolytes, whereas it decreases in the presence of urea. Values of *m* (for detail see Discussion) for all co-solutes obtained from the linear analysis of ΔΔ*G*_D_^o^ versus [co-solute] are given in Table 2. Following Mello and Barrick [37], the predicted ratio for non-methylamine-induced counteraction of urea’s effect on protein stability (for each of the protein) obtained by using the *m*-values of urea (*m*_u_) and non-methylamine osmolyte (counteracting osmolyte (*m*_CO_)) are also presented in Table 2. This table also shows the ratio of *m*_u_: *m*_CO_ required for thermodynamic counteraction. It is seen in Table 2 that myo-inositol exhibits counteraction at 2∶1 (urea: osmolyte) ratio for all the three proteins while ratio of urea-sorbitol and urea-taurine mixture varies depending on the protein chosen. We have further checked if the prediction ratio for the counteraction is indeed true by measuring heat-induced denaturation of each of the protein in the presence of the several concentrations of urea-non-methylamine mixtures at the ratio obtained from the predictions. Such heat-induced transition curves in the presence of the urea-non-methylamine mixtures are also presented in Figures 1 to 3, and the evaluated thermodynamic parameters are also given in Table 1. Figures 4 to 6 also show the plot of ΔΔ*G*_D_^o^ versus [urea-non-methylamine mixture], the molar concentrations of urea-non-methylamine mixtures. It is seen in these figures that the predicted ratio turns true for thermodynamic counteraction for urea-myo-inositol and urea-taurine mixtures. Interestingly, for all the proteins investigated, sorbitol shows no counteraction at the predicted ratio. The results indicate that, in addition to the methylamine, non-methylamine osmolytes, myo-inositol and taurine exhibit thermodynamic counteraction.

**Table 2:** *m*-values (dependence of Δ*G*_D_^o^ on concentrations of different co-solutes) and the ratios, [urea]: [CO] predicted for perfect counteraction using equation 5 for RNase-A, lysozyme and α-LA at pH 7.0 and 25°C.

	RNase-A		Lysozyme		α-LA	
Co-solute	*m* kcal mol^−1^ M^−1^	Predicted Ratio [urea]:[CO]	*m* kcal mol^−1^ M^−1^	Predicted Ratio [urea]:[CO]	*m* kcal mol^−1^ M^−1^	Predicted Ratio [urea]: [CO]
Urea	−1.07	–	−0.70	–	−0.54	–
Myo-inositol	2.15	2.0∶1.0	1.38	2.0∶1.0	1.09	2.0∶1.0
Sorbitol	1.81	2.0∶1.2	0.90	2.0∶1.6	1.08	2.0∶1.0
Taurine	1.41	2.0∶1.5	1.13	2.0∶1.2	1.08	2.0∶1.0
Betaine^a^	0.98	2.0∶2.2	2.33	2.0∶0.6	1.39	2.0∶0.8
GPC^b^	–	2.0∶2.7	–	–	–	–

a The values are taken from Singh *et al.* [69].

b Values taken from Burg *et al*. [70].

To validate our observations from thermodynamic measurements, we have carried out measurements of enzymatic parameters (*K*_m_ and *k*_cat_) of RNase-A and lysozyme in the absence and presence of urea, stabilizing osmolytes and their mixtures at pH 7.0 and 25°C. These results are shown in Table 3. Values of *K*_m_ and *k*_cat_ of both the enzymes in the absence of co-solutes are in agreement with the earlier reports [34], [38], [39] suggesting that the values obtained in this study are authentic and accurate. It is seen in Table 3 that (a) urea increases *K*_m_ and decreases *k*_cat_ of both enzymes, and (b) myo-inositol, sorbitol and taurine do not perturb *K*_m_ and *k*_cat_, i.e., they are compatible osmolytes. This table also shows values of kinetic parameters in the presence of non-methylamine-urea mixtures (at the predicted molar ratio of non-methylamine and urea). It is observed that (a) myo-inositol and taurine reverses the effect urea on *K*_m_ and *k*_cat_ of enzymes, (b) sorbitol does not show any counterbalancing effects on both the kinetic parameters of both enzymes.

**Table 3:** Activity parameters of RNase-A and lysozyme in the presence and absence of co-solutes at pH 7.0 and 25°C.^a^

	RNase-A			Lysozyme	
[Co-solute]	*K*_m_	*k*_cat_	[Co-solute]	*K*_m_	*k*_cat_
M	mM	s^−1^	M	µg ml^−1^	mg ml^−1^ s^−1^ M^−1^
*Urea*			*Urea*		
0.00	0.99±0.02	3.44±0.08	0.00	77.8±2	484.1±29
0.50	1.16±0.02	3.22±0.10	0.50	87.3±3	451.6±30
1.00	1.36±0.04	3.02±0.06	1.00	93.4±4	417.7±20
1.50	1.49±0.02	2.75±0.07	1.50	97.6±3	380.6±15
2.00	1.69±0.03	2.48±0.09	2.00	107.7±4	349.6±20
*Myo-inositol*			*Myo-inositol*		
0.10	1.00±0.04	3.50±0.18	0.10	77.9±2	483.5±35
0.20	0.99±0.04	3.45±0.14	0.20	77.1±3	482.9±30
0.30	1.01±0.09	3.44±0.23	0.30	76.5±2	484.5±15
0.40	1.02±0.06	3.47±0.13	0.40	77.8±1	484.9±20
*Sorbitol*			*Sorbitol*		
0.25	1.01±0.09	3.44±0.23	0.25	77.7±3	483.0±15
0.50	1.00±0.07	3.50±0.18	0.50	76.8±4	483.8±16
0.75	1.01±0.05	3.47±0.30	0.75	77.1±2	484.6±11
1.00	0.99±0.10	3.45±0.14	1.00	76.1±2	485.1±10
*Taurine*			*Taurine*		
0.10	1.00±0.05	3.47±0.08	0.10	78.3±3	484.9±18
0.20	0.99±0.02	3.46±0.09	0.20	77.8±3	483.1±20
0.30	0.97±0.05	3.43±0.10	0.30	77.4±1	484.5±16
0.40	0.95±0.02	3.41±0.06	0.40	77.1±1	483.6±09
*Myo-inositol+urea*			*Myo-inositol+urea*		
0.10+0.20	1.05±0.03	3.44±0.08	0.10+0.20	78.0±3	483.2±12
0.20+0.40	1.03±0.06	3.40±0.08	0.20+0.40	78.3±3	481.1±15
0.30+0.60	1.00±0.06	3.40±0.07	0.30+0.60	77.4±4	480.2±07
0.40+0.80	1.06±0.04	3.35±0.09	0.40+0.80	77.1±3	481.6±11
*Sorbitol+urea*			*Sorbitol+urea*		
0.30+0.50	1.17±0.02	3.20±0.10	0.40+0.50	87.5±2	448.2±12
0.60+1.00	1.33±0.04	3.05±0.06	0.80+1.00	93.1±3	435.6±14
0.90+1.50	1.45±0.02	2.70±0.07	1.20+1.50	96.3±2	378.0±07
1.20+2.00	1.70±0.03	2.50±0.09	1.60+2.00	107.2±4	345.9±15
*Taurine+urea*			*Taurine+urea*		
0.15+0.20	1.02±0.03	3.44±0.08	0.12+0.20	79.9±3	477.1±18
0.30+0.40	1.03±0.06	3.39±0.08	0.24+0.40	81.7±4	462.1±30
0.45+0.60	1.00±0.06	3.36±0.07			

a A ± with *K*_m_ and *k*_cat_ is the mean error obtained from the triplicate measurements.

## Discussion

The preceding section presents results of our measurements of the effects of non-methylamines (myo-inositol, sorbitol and taurine) and urea alone and in combination on structure, stability and function of three model proteins (RNase-A, lysozyme and α-LA). A question arises: Do these results have any relevance to kidney proteins? It has been shown that stabilizing osmolytes including non-methylamines are preferentially excluded from the protein domain [40], [41]. This property of stabilizing osmolytes has two important consequences. (a) There is no binding between osmolyles and proteins. (b) Stabilization by osmolytes is protein non-specific due to osmophobic effect originating from the overwhelming unfavourable interaction between osmolytes and the peptide backbone [41]. Hence, findings of this study will have relevance to kidney proteins as well.

Analysis of a thermal denaturation curve according to equation (1) assumes that denaturation follows a two-state mechanism. Differential scanning calorimetric (DSC) measurements suggest that this is, indeed, true for these proteins in the absence of osmolytes [42], [43]. Furthermore, DSC measurements suggest that the heat-induced denaturation of RNase-A, and lysozyme in the presence of methylamines [38], [44] and urea [45] is a two-state process. To check whether the two-state assumption is valid in the presence of kidney osmolytes, non-methylamines (myo-inositol, sorbitol and taurine) and urea-osmolyte mixtures, the heat-induced denaturation of RNase-A, lysozyme, and α-LA in the presence of 2.0 M urea, 0.4 M myo-inositol, 1.0 M sorbitol, 0.4 M taurine and their combined mixtures were measured by two different optical techniques, namely, mean residue ellipticity at 222 nm ([*θ*]_222_) which measures the change in the peptide backbone conformation (see Figures S1 to S3 in Appendix S1) and Δ*ε* in the near- UVregion (see Figures 1 to 3), which measures the change in the tyrosine/tryptophan environment. All denaturation curves were analyzed for Δ*H*_m_ and *T*_m_ using a non-linear analysis according to equation (1). We compared Δ*H*_m_ and *T*_m_ values thus obtained from Δ*ε* measurements with those from [*θ*]_222_ measurements. It is seen in Table 1 that both measurements gave, within experimental errors, identical values of these thermodynamic parameters. Thus, a two-state assumption for thermal denaturation of RNase-A, lysozyme, and α-LA in the presence of urea, stabilizing osmolytes and their predicted ratio combined mixtures seems to be valid.

It is necessary to show that values of Δ*C*_p_, Δ*H*_m_ and *T*_m_ obtained from the analysis of thermal transition curves such as shown in Figures 1 to 3, are in good agreement with those obtained from DSC measurements. Makhatadze and Privalov [45] have reported calorimetric values of these parameters for RNase-A and lysozyme in the absence and presence of 1 and 2 M urea at pH 5.5 and 4.6, respectively. Since these pH values are different from those used in this study, a comparison of Δ*H*_m_ and *T*_m_ are not possible. However, a direct comparison of Δ*C*_p_ values is possible, for Δ*C*_p_ is independent of pH [36]. It has been observed that there exists, within experimental errors, an excellent agreement between calorimetric Δ*C*_p_ values [45] and those observed in this study (see Tables S1 and S5 in Appendix S1).

To compare a thermodynamic quantity of a protein in the absence and presence of urea (0.2–2.0 M), stabilizing osmolytes (0.1–1.0 M) and their mixture, it is necessary to show that the structural characteristics of the two end states, i.e, N and D states of denaturations of RNase A, lysozyme and α-LA, are not affected due to the presence of urea, stabilizing osmolytes and their combined mixtures. We observed that the far- and near-UV CD spectra of both native and denatured proteins in the absence and presence of the osmolytes were, within the experimental errors, identical (see Figures S4 to S6 in Appendix S1). These observations on the native proteins are consistent with the x-ray results showing that the native structure of proteins is unperturbed by osmolyte [46] and with the size exclusion chromatography results suggesting that an osmolyte has no effect on the dimensions of the native proteins [47], [48], [49]. Our observation on denatured proteins is also in agreement with the report that methylamines are non-perturbing in its effects on fully solvent-exposed amide protons [50]. Hence, a comparison of a thermodynamic property of denaturation in the presence and absence of the urea, non-methylamine osmolytes and their mixtures is valid.

The thermodynamic basis of urea-osmolyte compensation on protein stability has been explained in terms of preferential binding and preferential exclusion of these co-solutes, respectively [51], which is supported by observations on the transfer-free energy of the protein groups from the solvent water to the co-solute solutions [52] . Based on this model, osmolytes stabilize proteins by shifting the denaturation equilibrium, N state ↔ D state toward the left, while urea destabilizes proteins by shifting the denaturation equilibrium toward the right, and urea-osmolyte mixture brings about compensatory effect. Thus, what effects co-solutes will have on the denaturation equilibrium, N state ↔ D state under the native condition will be known only by measuring Δ*G*_D_^o^ (value of Gibbs energy change, Δ*G*_D_ at 25°C) in different solvent conditions. Values of Δ*G*_D_^o^ of all proteins at different concentrations of urea and each stabilizing osmolyte are given in Table 1. These observations are also shown in Figures 4 to 6 which show the plots of ΔΔ*G*_D_^o^ versus [urea] and ΔΔ*G*_D_^o^ versus each [non-methylamine] (ΔΔ*G*_D_^o^ is defined as the difference between Δ*G*_D_^o^ in the presence of co-solute and Δ*G*_D_^o^ in the absence of co-solute). It is seen in Table 1 and Figures 4 to 6 that myo-inositol, sorbitol and taurine increase Δ*G*_D_^o^ of each protein while urea decreases Δ*G*_D_^o^ in a concentration dependent manner.

The main reason for the thermodynamic counteraction is that urea and its counteracting osmolytes act independently on the proteins, i.e., none of the co-solutes alters the efficacy of the other in forcing proteins to fold or unfold [52]. This means that the sensitivity of Δ*G*_D_^o^ of proteins on [urea] (the *m*_u_-value) is independent of [CO], the counteracting osmolyte (CO) concentration, and vice-versa. Following Mello and Barrick [37], the combined effects of urea and CO on denaturation free energies can be modeled as being linear in both co-solutes:
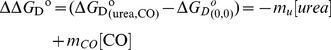
(5)where Δ*G*_D_^o^_(urea, CO)_ and Δ*G*_D_^o^_(0,0)_ are values of Δ*G*_D_^o^ measured in the presence and absence of urea- CO mixture, respectively, and *m*_co_ gives the dependence of Δ*G*_D_^o^_(CO)_ on [CO]. It then follows from equation (5) that the ratio [urea]: [CO] for a perfect compensation can be predicted if one knows values of *m*_u_ and *m*_co_. For a protein-co-solute system a linear analysis of ΔΔ*G*_D_^o^ (co-solute) versus [co-solute] plots (Figures 4 to 6) yielded the *m*-values (*m*_u_ and *m*_co_). These values are shown in Table 2. Values given in this table, suggest that for urea-myo-inositol interaction with proteins, the ratio *m*_co_: *m*_u_ is 2.0∶1.0 for all the proteins. This implies that a perfect counteraction is expected at 2∶1 ([urea]: [CO]) ratio. It is seen in Figure 4 that this is indeed true experimentally. We, therefore, conclude that urea-myo-inositol exhibits perfect counteraction at 2.0∶1.0, and these two co-solutes act on proteins independent of each other. Interestingly, the ratio of myo-inositol-induced counteraction of urea’s effect observed here on the three proteins is the same ratio found for methylamine-induced counteraction.

**Figure 7:**
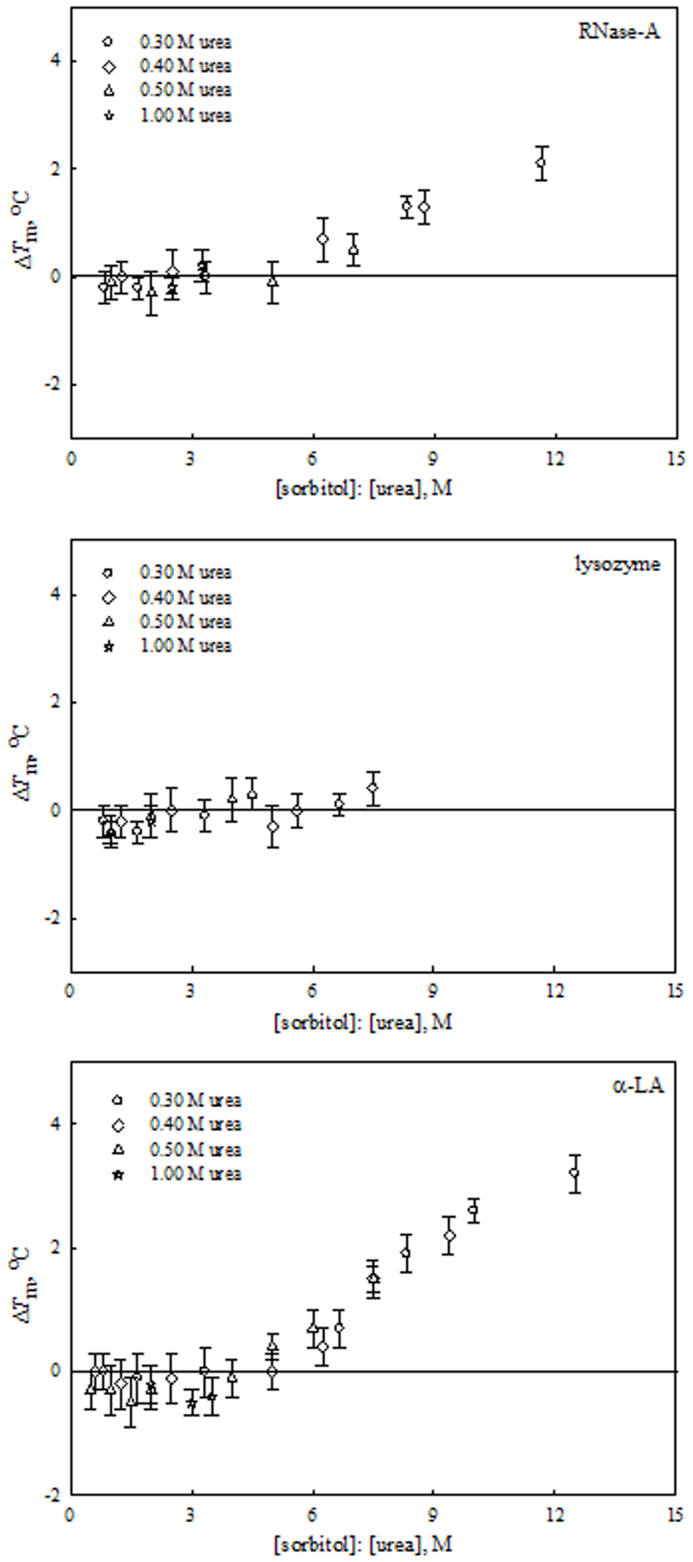
Effect of [sorbitol]: [urea] mixtures at different ratios on the stability of proteins at pH 7.0 and 25°C. Plots of Δ*T*_m_ versus different combined ratios of [sorbitol]: [urea] mixtures.

Similarly, from the measured *m*-values of urea and sorbitol (and taurine) for all proteins we predicted the [urea]: [sorbitol] (and [urea]: [taurine]) ratios for counteraction (see Table 2). Table 2 indicates that the predicted ratio for thermodynamic counteraction is apparently protein dependent for urea-sorbitol and urea-taurine mixtures. Figures 4 to 6 show plots of experimentally measured values of ΔΔ*G*_D_^o^ of urea, non-methylamines and the urea-non-methylamine mixtures at the given predicted molar ratios. To our surprise, the highly stabilizing osmolyte, sorbitol completely lost its ability to offset the effects of urea on all proteins studied (Figure 5). This observation on sorbitol led us to conclude that in spite of being a good protein folder in various stress conditions [34], [53], [54], it does not refold the urea-denatured proteins. To check whether it is, indeed, true at all sorbitol to urea ratios, we have measured heat-induced denaturation curves of each protein as function of [sorbitol] at 0.25 M and above in the presence of a fixed urea concentration in the range 0.3, 0.4, 0.5, and 1.0 M (e.g. see Figure S7 in Appendix S1). These results were analyzed for *T*_m_, and its values under different solvent conditions are shown in Table S13 in Appendix S1. For each protein effect of sorbitol-urea mixture on Δ*T*_m_ is shown in Figure 7, where Δ*T*_m_ = *T*_m_ in the presence of sorbitol-urea mixture – *T*_m_ in the presence of urea alone. It is seen in this figure that sorbitol has lost its ability to counteract the effect of urea on thermal stability of proteins if its concentration is six times less than that of urea. However, if [sorbitol]: [urea] ratio is ≥6, Δ*T*_m_ of RNase-A and α-LA increases with an increase in [sorbitol]: [urea] ratio, suggesting that sorbitol can refold urea-denatured proteins; about 2.1 and 3.2°C increase in *T*_m_ of RNase-A and α-LA at a ratio of 12, respectively. Due to experimental constraints, we could not measure Δ*T*_m_ values above 7.5 ([sorbitol]: [urea]) ratio.

We have seen above that myo-inositol has the ability to counteract the deleterious effect of urea on protein stability at a ratio of 0.5 for [myo-inositol]: [urea] (see Figure 4) whereas sorbitol completely fails to do so (see Figure 7). Is this difference in the counteraction of urea’s effects by sorbitol (linear 6-carbon polyol) and myo-inositol (cyclic 6-carbon polyol) due to difference in their structure? Our research is underway to answer this question.

Contrary to myo-inositol and sorbitol, taurine was found to have protein specific effect on counteracting the urea’s effect. At the predicted ratio (2.0∶1.5 for RNase-A and 2.0∶1.0 for α-LA) [urea]: [taurine] there exists a perfect counteraction of the urea’s effect on proteins. However, only a partial compensation is observed in the case of lysozyme at the predicted ratio of 2.0∶1.2 (see Figure 6). These observations on taurine clearly indicate that it exhibits protein specific counteraction. It is worth noting that, as observed in the case of taurine, methylamine osmolytes found in human kidney or in marine invertebrates, with a few exceptions, always yield partial counteraction [7], [55], [56], [57], [58], [59].

It may be noted that thermodynamic quantities obtained here are merely physical parameters and hence needs to be validated against the biological function. We have therefore, validated our observations on the thermodynamic parameters of the proteins by measuring functional activity parameters (*K*_m_ and *k*_cat_) of RNase-A and lysozyme. If the observed urea-osmolyte counteraction phenomenon is due to the shift in the thermodynamic equilibrium, N (functional) state ↔ D (non-functional) state (as indicated by Δ*G*_D_^o^ values), then it must be reflected in activity parameters of the enzymes. Because urea is known to push this thermodynamic equilibrium toward the right [51], [60] and consequently affects the number of enzymatically active fractions. As a result, *K*_m_ of the enzyme-catalyzed reaction is increased and *k*_cat_ is decreased in the presence of urea [7]. In agreement with this, results given in Table 3 show that *K*_m_ of both RNase-A and lysozyme increases while *k*_cat_ is decreased in urea’s presence. Furthermore, if the osmolytes myo-inositol, sorbitol and taurine have the same efficacy to fold urea-denatured proteins, then the number of active fractions is expected to be similar and consequently will have the same ability to counter the inactivation caused by urea on the enzymes. Our results given in Table 3 suggest that at predicted ratios myo-inositol and taurine can nullify the effect of urea inactivation of the enzymes in terms of *K*_m_ and *k*_cat_, while sorbitol has no ability to counterbalance the effects of urea on *K*_m_ and *k*_cat_ of both the enzymes. Thus, the observed effects on the activity parameters are in good agreement with those of the thermodynamic measurements. The data therefore, suggest that the effectiveness of refolding urea-denatured protein is different for all the three osmolytes. Myo-inositol is able to generate all of the urea-denatured protein fractions to functionally active ones while taurine shows ratio dependent counteraction. Sorbitol cannot refold urea-denatured proteins and hence it cannot convert the urea-inactivated protein fractions to functionally active molecules. Therefore, among the non-methylamines used in this study myo-inositol but not sorbitol or taurine stands as the best urea’s counteractant on protein stability and function.

Taken together, our results suggest that similar to the methylamine osmolytes, some non-methylamine osmolytes (especially myo-inositol and taurine) are good counteractants of urea’s effect on protein stability and function. Sorbitol is a key component of the renal cell osmotic system, but it may not be part of the urea-counteraction system as high concentrations of sorbitol have many clinical complications including, diabetes, cataract, neuropathy [61], [62].

As observed in our study, although myo-inositol is a better counteractant of urea’s effect on protein stability and function than methylamines, the major response of renal cells in cultures to high urea is the increase of GPC, not myo-inositol [63]. It is very likely that evolution might have favored GPC over myo-inositol to counteract the deleterious effects of urea in the renal cells. However, we do not rule out the possibility that myo-inositol (or taurine) might be acting as a counteracting osmolyte in other organisms wherein GPC or other methylamines are absent.

Inside the cells the urea-counteraction system is not confined to only one. There exist multiple urea-counteracting systems. The most efficient one (based on this study) is the urea-myo-inositol system and other includes urea-taurine and urea-methylamine systems that work in a protein specific manner. Speculatively, the urea-myo-inositol may act as a house keeping counteracting system while the other two may invoke only on cellular demands (e.g., extreme stresses) because the existing levels of myo-inositol may not be sufficient enough to take care of the whole proteome stability under high hypertonic conditions (e.g., anti-diuretic condition) in the kidney. Interestingly, almost all reports on the osmotic content of the kidney cells were derived from the measurements under high hypertonic conditions [64], [65], [66], [67], [68]. It is, therefore, important to estimate osmotically active solute in the kidney cells under hypotonic conditions or diuresis.

## Supporting Information

Appendix S1 Supplementary data. The following supplementary data is available: **FIGURE S1.** Effect of urea, osmolyte and their predicted ratio mixtures on RNase-A; **FIGURE S2.** Effect of urea, osmolyte and their predicted ratio mixtures on Lysozyme; **FIGURE S3.** Effect of urea, osmolyte and their predicted ratio mixtures on α-LA; **FIGURE S4.** Effect of urea, myo-inositol and their predicted ratio mixtures on secondary and tertiary structures of proteins; **FIGURE S5.** Effect of urea, sorbitol and their predicted ratio mixtures on secondary and tertiary structures of proteins; **FIGURE S6.** Effect of urea, taurine and their predicted ratio mixtures on secondary and tertiary structures of proteins; **FIGURE S7.** Effect of urea, sorbitol and their mixtures (at different ratios) on the proteins at pH 7.0; **Table S1.** Thermodynamic parameters associated with the thermal unfolding of RNase-A at different pH values in the presence and absence of urea; **Table S2.** Thermodynamic parameters associated with the thermal unfolding of RNase-A at different pH values in the presence and absence of myo-inositol; **Table S3.** Thermodynamic parameters associated with the thermal unfolding of RNase-A at different pH values in the presence and absence of sorbitol; **Table S4.** Thermodynamic parameters associated with the thermal unfolding of RNase-A at different pH values in the presence and absence of taurine; **Table S5.** Thermodynamic parameters associated with the thermal unfolding of lysozyme at different pH values in the presence and absence of urea; **Table S6.** Thermodynamic parameters associated with the thermal unfolding of lysozyme at different pH values in the presence and absence of myo-inositol; **Table S7.** Thermodynamic parameters associated with the thermal unfolding of lysozyme at different pH values in the presence and absence of sorbitol; **Table S8.** Thermodynamic parameters associated with the thermal unfolding of lysozyme at different pH values in the presence and absence of taurine; **Table S9.** Thermodynamic parameters associated with the thermal unfolding of α-LA at different pH values in the presence and absence of urea; **Table S10.** Thermodynamic parameters associated with the thermal unfolding of α-LA at different pH values in the presence and absence of myo-inositol; **Table S11.** Thermodynamic parameters associated with the thermal unfolding of α-LA at different pH values in the presence and absence of sorbitol; **Table S12.** Thermodynamic parameters associated with the thermal unfolding of α-LA at different pH values in the presence and absence of taurine; **Table S13.** Thermal denaturation studies on proteins to see the counteracting effect of sorbitol with increasing urea concentration at pH 7.0.
(DOC)

## References

[1] Wolff SD, Balaban RS (1990) Regulation of the predominant renal medullary organic solutes in vivo. Annu Rev Physiol 52: 727–746. 2184774 10.1146/annurev.ph.52.030190.003455

[2] MacMillen RE, Lee AK (1967) Australian desert mice: independence of exogenous water. Science 158: 383–385. 6061891 10.1126/science.158.3799.383

[3] Santos BC, Chevaile A, Hebert MJ, Zagajeski J, Gullans SR (1998) A combination of NaCl and urea enhances survival of IMCD cells to hyperosmolality. Am J Physiol 274: F1167–1173. 9841510 10.1152/ajprenal.1998.274.6.F1167

[4] Michea L, Ferguson DR, Peters EM, Andrews PM, Kirby MR, et al (2000) Cell cycle delay and apoptosis are induced by high salt and urea in renal medullary cells. Am J Physiol Renal Physiol 278: F209–218. 10662725 10.1152/ajprenal.2000.278.2.F209

[5] Nozaki Y, Tanford C (1963) The Solubility of Amino Acids and Related Compounds in Aqueous Urea Solutions. J Biol Chem 238: 4074–4081. 14086747

[6] Yancey PH, Somero GN (1979) Counteraction of urea destabilization of protein structure by methylamine osmoregulatory compounds of elasmobranch fishes. Biochem J 183: 317–323. 534499 10.1042/bj1830317 PMC1161561

[7] Yancey PH, Somero GN (1980) Methylamine osmoregulatory compounds in elasmobranch fishes reverse urea inhibition of enzymes. J Exp Zool 212: 205–213.

[8] Auton M, Bolen DW (2005) Predicting the energetics of osmolyte-induced protein folding/unfolding. Proc Natl Acad Sci U S A 102: 15065–15068. 16214887 10.1073/pnas.0507053102 PMC1257718

[9] Kraus ML, Kraus AP (2001) Carbomylation of amino acids and proteins in uremia. Kidney Int 78: 102–107. 10.1046/j.1523-1755.2001.59780102.x 11168993

[10] Nystrom T (2005) Role of oxidative carbonylation in protein quality control and senescence. EMBO J 24: 1311–1317. 15775985 10.1038/sj.emboj.7600599 PMC1142534

[11] Schmolke M, Bornemann A, Guder WG (1991) Distribution and regulation of organic osmolytes along the nephron. Contrib Nephrol 95: 255–263. 1807916 10.1159/000420667

[12] Yancey PH, Burg MB (1989) Distribution of major organic osmolytes in rabbit kidneys in diuresis and antidiuresis. Am J Physiol 257: F602–607. 2801962 10.1152/ajprenal.1989.257.4.F602

[13] Garcia-Perez A, Burg MB (1991) Renal medullary organic osmolytes. Physiol Rev 71: 1081–1115. 1924548 10.1152/physrev.1991.71.4.1081

[14] Schmolke M, Schilling A, Keiditsch E, Guder WG (1996) Intrarenal distribution of organic osmolytes in human kidney. Eur J Clin Chem Clin Biochem 34: 499–501. 8831052

[15] Schmolke M, Bornemann A, Guder WG (1996) Site-specific regulation of organic osmolytes along the rat nephron. Am J Physiol 271: F645–652. 8853427 10.1152/ajprenal.1996.271.3.F645

[16] Guder WG, Beck FX, Schmolke M (1990) Regulation and localization of organic osmolytes in mammalian kidney. Klin Wochenschr 68: 1091–1095. 2280574 10.1007/BF01798058

[17] Schmolke M, Guder WG (1989) Metabolic regulation of organic osmolytes in tubules from rat renal inner and outer medulla. Ren Physiol Biochem 12: 347–358. 2623349 10.1159/000173212

[18] Wirthensohn G, Lefrank S, Schmolke M, Guder WG (1989) Regulation of organic osmolyte concentrations in tubules from rat renal inner medulla. Am J Physiol 256: F128–135. 2912156 10.1152/ajprenal.1989.256.1.F128

[19] von Recklinghausen IR, Scott DM, Jans AW (1991) An NMR spectroscopic characterization of a new epithelial cell line, TALH-SVE, with properties of the renal medullary thick ascending limb of Henle’s loop. Biochim Biophys Acta 1091: 179–187. 1847303 10.1016/0167-4889(91)90059-7

[20] Ruhfus B, Kinne RK (1996) Hypotonicity-activated efflux of taurine and myo-inositol in rat inner medullary collecting duct cells: evidence for a major common pathway. Kidney Blood Press Res 19: 317–324. 8990043 10.1159/000174094

[21] Stokes JB, Grupp C, Kinne RK (1987) Purification of rat papillary collecting duct cells: functional and metabolic assessment. Am J Physiol 253: F251–262. 3303974 10.1152/ajprenal.1987.253.2.F251

[22] Lee RE (1991) Principles of insect low temperature tolerance. In: Lee, Jr, RE, Denlinger, DL (Eds), Insects at Low Temperature Chapman and Hall, New York and London: 17–46.

[23] Nakanishi T, Turner RJ, Burg MB (1989) Osmoregulatory changes in myo-inositol transport by renal cells. Proc Natl Acad Sci U S A 86: 6002–6006. 2762310 10.1073/pnas.86.15.6002 PMC297760

[24] Mahler S, Kinne-Saffran E, Fujisue H, Kinne RK, Follmann W (1998) Regulation of sorbitol content in cultured porcine urinary bladder epithelial cells. Am J Physiol 274: F342–347. 9486229 10.1152/ajprenal.1998.274.2.F342

[25] Nakanishi T, Uyama O, Nakahama H, Takamitsu Y, Sugita M (1993) Determinants of relative amounts of medullary organic osmolytes: effects of NaCl and urea differ. Am J Physiol 264: F472–F479. 8456960 10.1152/ajprenal.1993.264.3.F472

[26] Yancey PH (2003) Proteins and counteracting osmolytes. Biologist 50: 126–131.

[27] Bigelow CC, Geschwind II (1960) Difference specta of amino acids and proteins in aqueous media of high refractive index. C R Trav Lab Carlsberg 31: 283–304. 13800628

[28] Hamaguchi K, Kurono A (1968) Structure of Muramidase (lysozyme) III. Effect of 2-chloroethanol, ethanol and dioxane on the stability of Muramidase. J Biochem 54: 497–505. 10.1093/oxfordjournals.jbchem.a127822 14099005

[29] Sugai S, Yashiro H, Nitta K (1973) Equilibrium and kinetics of the unfolding of alpha-lactalbumin by guanidine hydrochloride. Biochim Biophys Acta 328: 35–41. 4761990 10.1016/0005-2795(73)90327-9

[30] Pace CN (1986) Determination and analysis of urea and guanidine hydrochloride denaturation curves. Methods Enzymol 131: 266–280. 3773761 10.1016/0076-6879(86)31045-0

[31] Sinha A, Yadav S, Ahmad R, Ahmad F (2000) A possible origin of differences between calorimetric and equilibrium estimates of stability parameters of proteins. Biochem J 345 Pt 3: 711–717. PMC1220808 10642532

[32] Yadav S, Ahmad F (2002) A new method for the determination of stability parameters of proteins from their heat-induced denaturation curves. Anal Biochem 283: 207–213. 10.1006/abio.2000.4641 10906241

[33] Maurel CR, Westmoreland DG (1976) Catalytic implications of electrostatic potential: The lytic activity of lysozyme as a model. J Mol Biol 102: 253–264. 5609 10.1016/s0022-2836(76)80052-6

[34] Haque I, Singh R, Ahmad F, Moosavi-Movahedi AA (2005) Testing polyols’ compatibility with Gibbs energy of stabilization of proteins under conditions in which they behave as compatible osmolytes. FEBS Lett 579: 3891–3898. 15990095 10.1016/j.febslet.2005.06.005

[35] Singh R, Haque I, Ahmad F (2005) Counteracting osmolyte trimethylamine N-oxide destabilizes proteins at pH below its pKa. Measurements of thermodynamic parameters of proteins in the presence and absence of trimethylamine N-oxide. J Biol Chem 280: 11035–11042. 15653673 10.1074/jbc.M410716200

[36] Becktel WJ, Schellman JA (1987) Protein stability curves. Biopolymers 26: 1859–1877. 3689874 10.1002/bip.360261104

[37] Mello CC, Barrick D (2003) Measuring the stability of partly folded proteins using TMAO. Protein Sci 12: 1522–1529. 12824497 10.1110/ps.0372903 PMC2323936

[38] Santoro MM, Liu Y, Khan SM, Hou LX, Bolen DW (1992) Increased thermal stability of proteins in the presence of naturally occurring osmolytes. Biochemistry 31: 5278–5283. 1376620 10.1021/bi00138a006

[39] Maurel P, Douzou P (1976) Catalytic implications of electrostatic potentials: the lytic activity of lysozymes as a model. J Mol Biol 102: 253–264. 5609 10.1016/s0022-2836(76)80052-6

[40] Bolen DW, Baskakov IV (2001) The osmophobic effect: natural selection of a thermodynamic force in protein folding. J Mol Biol 310: 955–963. 11502004 10.1006/jmbi.2001.4819

[41] Singh LR, Poddar NK, Dar TA, Rahman S, Kumar R, et al (2011) Forty Years of Research on Osmolyte-Induced Protein Folding and Stability. Journal of the Iranian Chemical Society 8: 1–23.

[42] Privalov PL (1979) Stability of proteins: small globular proteins. Adv Protein Chem 33: 167–241. 44431 10.1016/s0065-3233(08)60460-x

[43] Pfeil W, Sadowski ML (1985) A scanning calorimetric study of bovine and human apo-a-lactalbumin. Stud Biophys 109: 163–170.

[44] Plaza del Pino IM, Sanchez-Ruiz JM (1995) An osmolyte effect on heat capacity change for protein folding. Biochemistry 34: 8621–8630. 7542026 10.1021/bi00027a011

[45] Makhatadze GI, Privalov PL (1992) Protein interactions with urea and guanidinium chloride. A calorimetric study. J Mol Biol 226: 491–505. 1322462 10.1016/0022-2836(92)90963-k

[46] Ratnaparkhi GS, Varadarajan R (2001) Osmolytes stabilize ribonuclease S by stabilizing its fragments S protein and S peptide to compact folding-competent states. J Biol Chem 276: 28789–28798. 11373282 10.1074/jbc.M101906200

[47] Baskakov I, Bolen DW (1998) Forcing thermodynamically unfolded proteins to fold. J Biol Chem 273: 4831–4834. 9478922 10.1074/jbc.273.9.4831

[48] Baskakov I, Wang A, Bolen DW (1998) Trimethylamine-N-oxide counteracts urea effects on rabbit muscle lactate dehydrogenase function: a test of the counteraction hypothesis. Biophys J 74: 2666–2673. 9591690 10.1016/S0006-3495(98)77972-X PMC1299606

[49] Qu Y, Bolen CL, Bolen DW (1998) Osmolyte-driven contraction of a random coil protein. Proc Nat Acad Sci U S A 95: 9268–9273. 10.1073/pnas.95.16.9268 PMC21327 9689069

[50] Youxing Q, Bolen DW (2003) Hydrogen exchange kinetics of RNase-A and the urea: TMAO paradigm. Biochemistry 42: 5837–5849. 12741842 10.1021/bi0206457

[51] Timasheff SN (2002) Protein-solvent preferential interactions, protein hydration, and the modulation of biochemical reactions by solvent components. Proc Natl Acad Sci U S A 99: 9721–9726. 12097640 10.1073/pnas.122225399 PMC124992

[52] Holthauzen LMF, Bolen DW (2007) Mixed osmolytes: the degree to which one osmolyte affects the protein stabilizing ability of another. Protein Sci 16: 293–298. 17189473 10.1110/ps.062610407 PMC2203298

[53] Kaushik JK, Bhat R (1998) Thermal stability of proteins in aqueous polyol solutions. J Phys Chem B 102: 7058–7066.

[54] Haque I, Singh R, Moosavi-Movahedi AA, Ahmad F (2005) Effect of polyol osmolytes on DeltaG(D), the Gibbs energy of stabilisation of proteins at different pH values. Biophys Chem 117: 1–12. 15905020 10.1016/j.bpc.2005.04.004

[55] Burg MB, Kwon ED, Peters EM (1996) Glycerophosphocholine and betaine counteract the effect of urea on pyruvate kinase. Kidney Int Suppl 57: S100–104. 8941929

[56] Yancey PH, Somero GN (1978) Urea-requiring lactate dehydrogenases of marine elasmobranch fishes. J Comp Physiol 125: 135–141.

[57] Mashino T, Fridovich I (1987) Effects of urea and trimethylamine-N-oxide on enzyme activity and stability. Arch Biochem Biophys 258: 356–360. 3674879 10.1016/0003-9861(87)90355-9

[58] De Meis L, Inesi G (1998) Effects of organic solvents, methylamines, and urea on the affinity for Pi of the Ca2+- ATPase of sarcoplasmic reticulum. J Biol Chem 263: 157–161. 2961744

[59] Singh LR, Ali Dar T, Haque I, Anjum F, Moosavi-Movahedi AA, et al (2007) Testing the paradigm that the denaturing effect of urea on protein stability is offset by methylamines at the physiological concentration ratio of 2:1 (urea:methylamines). Biochim Biophys Acta 1774: 1555–1562. 17962089 10.1016/j.bbapap.2007.09.006

[60] Singh LR, Dar TA, Rahman S, Jamal S, Ahmad F (2009) Glycine betaine may have opposite effects on protein stability at high and low pH values. Biochimica et Biophysica Acta 1794: 929–935. 19254782 10.1016/j.bbapap.2009.02.005

[61] Green DA, Lattimer SA, Sima AF (1987) Sorbitol, phosphoinositidesk and sodium-pottasium-ATPase in the pathogenesis of diabetic complications. N Engl J Med 316: 599–564. 3027558 10.1056/NEJM198703053161007

[62] Burg MB, Kador PF (1988) Sorbitol, osmoregulation, and the complications of diabetes. J Clin Invest 81: 635–640. 3278002 10.1172/JCI113366 PMC442508

[63] Grunewald RW, Kinne RK (1999) Osmoregulation in the mammalian kidney: the role of organic osmolytes. J Exp Zool 283: 708–724. 10222592 10.1002/(sici)1097-010x(19990601)283:7<708::aid-jez9>3.0.co;2-v

[64] Hammerman MR, Sacktor B, Daughaday WH (1980) myo-Inositol transport in renal brush border vesicles and it inhibition by D-glucose. Am J Physiol 239: F113–120. 6773422 10.1152/ajprenal.1980.239.2.F113

[65] Bagnasco S, Balaban R, Fales HM, Yang YM, Burg M (1986) Predominant osmotically active organic solutes in rat and rabbit renal medullas. J Biol Chem 261: 5872–5877. 3700377

[66] Pehling G, Ullrich KJ (1956) [Occurrence of phosphorus compounds in various kidney sections and changes of their concentration in relation to diuretic conditions]. Pflugers Arch 262: 551–561. 10.1007/BF00362117 13359098

[67] Schimassek H, Kohl D, Bucher T (1959) Glycerylphosphorylcholin, die nierensubstanz “ma-mark” von Ullrich. Biochem Z 331: 87–97.

[68] Hutton JC, Schofield PJ, Williams JF, Hollows FC (1975) The localisation of sorbitol pathway activity in the rat renal cortex and its relationship to the pathogenesis of the renal complications of diabetes mellitus. Aust J Exp Biol Med Sci 53: 49–57. 125079 10.1038/icb.1975.5

[69] Singh LR, Dar TA, Rahman S, Jamal S, Ahmad F (2009) Glycine betaine may have opposite effects on protein stability at high and low pH values. Biochim Biophys Acta 1794: 929–935. 19254782 10.1016/j.bbapap.2009.02.005

[70] Burg MB, Peters EM (1998) Effects of glycine betaine and glycerophosphocholine on thermal stability of ribonuclease. Am J Physiol Renal Physiol 274: F762–F765. 10.1152/ajprenal.1998.274.4.F762 9575901

