# Supplementary material for: Testing the Ability of Non-Methylamine Osmolytes Present in Kidney Cells to Counteract the Deleterious Effects of Urea on Structure, Stability and Function of Proteins
Source: PLoS One. 2013 Sep 9;8(9):e72533. doi: 10.1371/journal.pone.0072533 (PMC3767660; doi:10.1371/journal.pone.0072533)
Supplement: Appendix S1 — Supplementary data. The following supplementary data is available: FIGURE S1. Effect of urea, osmolyte and their predicted ratio mixtures on RNase-A; FIGURE S2. Effect of urea, osmolyte and their predicted ratio mixtures on Lysozyme; FIGURE S3. Effect of urea, osmolyte and their predicted ratio mixtures on α-LA; FIGURE S4. Effect of urea, myo-inositol and their predicted ratio mixtures on secondary and tertiary structures of proteins; FIGURE S5. Effect of urea, sorbitol and their predicted ratio mixtures on secondary and tertiary structures of proteins; FIGURE S6. Effect of urea, taurine and their predicted ratio mixtures on secondary and tertiary structures of proteins; FIGURE S7. Effect of urea, sorbitol and their mixtures (at different ratios) on the proteins at pH 7.0; Table S1. Thermodynamic parameters associated with the thermal unfolding of RNase-A at different pH values in the presence and absence of urea; Table S2. Thermodynamic parameters associated with the thermal unfolding of RNase-A at different pH values in the presence and absence of myo-inositol; Table S3. Thermodynamic parameters associated with the thermal unfolding of RNase-A at different pH values in the presence and absence of sorbitol; Table S4. Thermodynamic parameters associated with the thermal unfolding of RNase-A at different pH values in the presence and absence of taurine; Table S5. Thermodynamic parameters associated with the thermal unfolding of lysozyme at different pH values in the presence and absence of urea; Table S6. Thermodynamic parameters associated with the thermal unfolding of lysozyme at different pH values in the presence and absence of myo-inositol; Table S7. Thermodynamic parameters associated with the thermal unfolding of lysozyme at different pH values in the presence and absence of sorbitol; Table S8. Thermodynamic parameters associated with the thermal unfolding of lysozyme at different pH values in the presence and absence of taurine; Table S9. Thermodynamic parame [file pone.0072533.s001.doc]

**-Supplementary data**

**Testing the Ability of Non-methylamine Osmolytes present in Kidney Cells to counteract the deleterious effects of Urea on Structure, Stability and Function of Proteins**

Sheeza Khan^1^, Zehra Bano^1^, Laishram R. Singh^2^, Md. Imtaiyaz Hassan^1^, Asimul Islam^1^, and Faizan Ahmad^1^

*^1^ Centre for Interdisciplinary Research in Basic Sciences, Jamia Millia Islamia, New Delhi-110025, India*

*^2^Dr. B. R. Ambedkar Center for Biomedical Research, University of Delhi, Delhi-110007, India*

To whom correspondence should be addressed.

Tel.: 91-11-26981733; Fax: 91-11-2698-3409

Email: fahmad@jmi.ac.in

**Table S1.** **Thermodynamic parameters associated with the thermal unfolding of RNase-A at different pH values in the presence and absence of urea.**

| **pH** | **[urea]**  M | **Δ*G*_D_^o^**  kcal mol^-1^ | ***T*_m_**  ^o^C | **Δ*H*_m_**  kcal mol^-1^ |
| --- | --- | --- | --- | --- |
| **pH 7.0** | 0.00  0.20  0.40  0.50  0.60  0.80 | 10.41 ± 0.14  10.19 ± 0.09  9.90 ± 0.18  9.93 ± 0.20  9.78 ± 0.16  9.56 ± 0.04 | 63.0 ± 0.2  62.5 ± 0.3  61.8 ± 0.2  61.6 ± 0.2  61.1 ± 0.3  60.4 ± 0.3 | 116 ± 2  115 ± 2  114 ± 3  114 ± 3  114 ± 3  113 ± 2 |
| **pH 6.0** | 1.00  1.50  2.00  0.00  0.20  0.40  0.50  0.60  0.80  1.00  1.50  2.00 | 9.28 ± 0.39  8.72 ± 0.24  8.34 ± 0.24  10.10 ± 0.14  9.87 ± 0.20  9.57 ± 0.29  9.59 ± 0.20  9.42 ± 0.16  9.18 ± 0.27  8.94 ± 0.18  8.39 ± 0.15  7.81 ± 0.24 | 60.0 ± 0.2  57.9 ± 0.3  56.3 ± 0.3  (56.0 ± 0.4)*  62.5 ± 0.3  61.9 ± 0.3  61.1 ± 0.4  60.9 ± 0.3  60.2 ± 0.2  59.4 ± 0.3  58.6 ± 0.3  56.6 ± 0.2  54.4 ± 0.3 | 111 ± 5  109 ± 4  108 ± 4  (109 ± 5)  114 ±2  113 ±3  112 ±4  112 ±3  111 ±3  111 ±4  110 ±3  108 ±3  106 ±4 |
| **pH 5.0** | 0.00  0.20  0.40  0.50  0.60  0.80  1.00  1.50  2.00 | 9.31 ± 0.14  9.02 ± 0.20  8.83 ± 0.28  8.70 ± 0.20  8.60 ± 0.16  8.45 ± 0.26  8.11 ± 0.18  7.56 ± 0.24  7.09 ± 0.23 | 61.0 ± 0.2  60.0 ± 0.4  59.2 ± 0.2  58.7 ± 0.2  58.4 ± 0.3  57.5 ± 0.3  56.6 ± 0.3  54.5 ± 0.4  52.8 ± 0.3 | 109 ± 2  108 ± 3  108 ± 4  107 ± 3  107 ± 3  107 ± 4  105 ± 3  103 ± 4  101 ± 4 |
| **pH 4.0** | 0.00  0.20  0.40  0.50  0.60  0.80  1.00  1.50  2.00 | 7.47 ± 0.13  7.23 ± 0.19  6.94 ± 0.26  6.81 ± 0.19  6.71 ± 0.16  6.55 ± 0.24  6.19 ± 0.17  5.62 ± 0.22  5.03 ± 0.20 | 55.8 ± 0.4  55.0 ± 0.2  54.0 ± 0.2  53.5 ± 0.3  53.1 ± 0.3  52.1 ± 0.4  51.4 ± 0.3  48.9 ± 0.3  46.3 ± 0.2 | 99 ± 2  98 ± 3  97 ± 4  96 ± 3  96 ± 3  96 ± 4  93 ± 3  91 ± 4  89 ± 4 |
| **pH 3.0** | 0.00  0.20  0.40  0.50  0.60  0.80  1.00  1.50  2.00 | 4.75 ± 0.22  4.49 ± 0.14  4.24 ± 0.19  4.18 ± 0.13  3.99 ± 0.12  3.76 ± 0.16  3.48 ± 0.21  2.86 ± 0.14  2.32 ± 0.14 | 44.0 ± 0.2  43.3 ± 0.4  42.4 ± 0.3  42.0 ± 0.2  41.4 ± 0.4  40.5 ± 0.2  39.8 ± 0.3  37.3 ± 0.4  34.8 ± 0.3 | 91 ± 4  89 ± 3  88 ± 4  88 ± 3  87 ± 3  86 ± 4  83 ± 5  80 ± 4  79 ± 5 |
| **pH 2.0** | 0.00  0.20  0.40  0.50  0.60  0.80  1.00  1.50  2.00 | 2.64 ± 0.13  2.36 ± 0.15  2.16 ± 0.11  2.03 ± 0.13  1.97 ± 0.13  1.77 ± 0.10  1.51 ± 0.11  1.08 ± 0.10  0.70±0.10 | 36.0 ± 0.3  35.0 ± 0.4  34.2 ± 0.3  33.7 ± 0.2  33.4 ± 0.2  32.7 ± 0.4  31.7 ± 0.4  29.8 ± 0.3  28.2±0.2 | 81 ± 4  79 ± 5  78 ± 4  77 ± 5  77 ± 5  75 ± 4  73 ± 5  71 ± 5  68±5 |
|  | **[urea]**  M | **Δ*C*_p_**  kcal mol^-1^ K^-1^ |  |  |
|  | 0.00  0.20  0.40  0.50  0.60  0.80  1.00  1.50  2.00 | 1.21 ± 0.04  1.22 ± 0.06  1.25 ± 0.07  1.22 ± 0.06  1.25 ± 0.08  1.25 ± 0.08  1.25 ± 0.07  1.25 ± 0.09  1.25 ± 0.09 |  |  |

*Values given in parentheses are from CD measurements.

**Table S2. Thermodynamic parameters associated with the thermal unfolding of RNase-A at different pH values in the presence and absence of myo-inositol.**

| **pH** | **[myo-inositol]**  M | **Δ*G*_D_^o^**  kcal mol^-1^ | ***T*_m_**  ^o^C | **Δ*H*_m_**  kcal mol^-1^ |
| --- | --- | --- | --- | --- |
| **pH 7.0** | 0.00  0.10  0.20  0.30  0.40 | 10.41 ± 0.14  10.67 ± 0.32  10.93 ± 0.32  11.05 ± 0.16  11.29 ± 0.32 | 63.0 ± 0.2  63.7 ± 0.2  64.3 ± 0.3  65.0 ± 0.3  65.7 ± 0.2  (66.0 ±0.4)* | 116 ± 2  117 ± 4  118 ± 4  118 ± 3  119 ± 4  (118 ± 3) |
| **pH 6.0** | 0.00  0.10  0.20  0.30  0.40 | 10.10 ± 0.14  10.34 ± 0.21  10.58 ± 0.32  10.80 ± 0.16  11.02 ± 0.18 | 62.5 ± 0.3  63.1 ± 0.3  63.6 ± 0.4  64.2 ± 0.2  64.8 ± 0.3 | 114 ± 2  115 ± 3  116 ± 4  117 ± 3  118 ± 3 |
| **pH 5.0** | 0.00  0.10  0.20  0.30  0.40 | 9.31 ± 0.14  9.53 ± 0.20  9.78 ± 0.20  9.99 ± 0.27  10.20 ± 0.18 | 61.0 ± 0.2  61.6 ± 0.4  62.2 ± 0.2  62.8 ± 0.3  63.4 ± 0.2 | 109 ± 2  110 ± 3  111 ± 3  112 ± 4  113 ± 3 |
| **pH 4.0** | 0.00  0.10  0.20  0.30  0.40 | 7.47 ± 0.13  7.69 ± 0.19  8.01 ± 0.20  8.21 ± 0.26  8.42 ± 0.28 | 55.8 ± 0.4  56.5 ± 0.2  57.1 ± 0.2  57.7 ± 0.3  58.4 ± 0.2 | 99 ± 2  100 ± 3  102 ± 3  103 ± 4  104 ± 4 |
| **pH 3.0** | 0.00  0.10  0.20  0.30  0.40 | 4.75 ± 0.21  4.93 ± 0.27  5.27 ± 0.22  5.54 ± 0.28  5.76 ± 0.22 | 44.0 ± 0.2  44.6 ± 0.2  45.6 ± 0.3  46.6 ± 0.4  47.4 ± 0.2 | 91 ± 4  92 ± 5  94 ± 4  95 ± 5  96 ± 4 |
| **pH 2.0** | 0.00  0.10  0.20  0.30  0.40 | 2.64 ± 0.13  2.81 ± 0.17  3.12 ± 0.19  3.33 ± 0.19  3.48 ± 0.16 | 36.0 ± 0.3  36.6 ± 0.4  37.5 ± 0.3  38.3 ± 0.2  39.0 ± 0.2 | 81 ± 4  82 ± 5  85 ± 5  86 ± 5  86 ± 4 |
|  | **[myo-inositol]**  M | **Δ*C*_p_**  kcal mol^-1^ K^-1^ |  |  |
|  | 0.00  0.10  0.20  0.30  0.40 | 1.21 ± 0.04  1.20 ± 0.06  1.18 ± 0.06  1.18 ± 0.08  1.18 ± 0.07 |  |  |

*Values given in parentheses are from CD measurements.

**Table S3. Thermodynamic parameters associated with the thermal unfolding of RNase-A at different pH values in the presence and absence of sorbitol.**

| **pH** | **[sorbitol]**  M | **Δ*G*_D_^o^**  kcal mol^-1^ | ***T*_m_**  ^o^C | **Δ*H*_m_**  kcal mol^-1^ |
| --- | --- | --- | --- | --- |
| **pH 7.0** | 0.00  0.25  0.50  0.75  1.00 | 10.41 ± 0.14  10.93 ± 0.14  11.36 ± 0.14  11.73 ± 0.23  12.27 ± 0.21 | 63.0 ± 0.2  64.3 ± 0.4  65.4 ± 0.3  66.9 ± 0.1  68.5 ± 0.2  (68.1 ± 0.2)* | 116 ± 2  118 ± 2  120 ± 3  121 ± 3  123 ± 3  (122 ± 4) |
| **pH 6.0** | 0.00  0.25  0.50  0.75  1.00 | 10.10 ± 0.14  10.61 ± 0.25  11.03 ± 0.28  11.60 ± 0.23  12.11 ± 0.21 | 62.5 ± 0.3  63.8 ± 0.2  64.9 ± 0.3  66.1 ± 0.4  67.5 ± 0.2 | 114 ± 2  116 ± 3  118 ± 4  121 ± 3  123 ± 3 |
| **pH 5.0** | 0.00  0.25  0.50  0.75  1.00 | 9.31 ± 0.14  9.88 ± 0.25  10.17 ± 0.27  10.43 ± 0.23  10.82 ± 0.33 | 61.0 ± 0.2  62.1 ± 0.4  63.2 ± 0.3  64.8 ± 0.2  66.4 ± 0.3 | 109 ± 2  112 ± 3  113 ± 4  113 ± 3  114 ± 4 |
| **pH 4.0** | 0.00  0.25  0.50  0.75  1.00 | 7.47 ± 0.13  7.88 ± 0.23  8.25 ± 0.26  8.69 ± 0.22  9.17 ± 0.31 | 55.8 ± 0.4  56.9 ± 0.4  58.0 ± 0.2  59.4 ± 0.3  61.0 ± 0.2 | 99 ± 2  101 ± 3  103 ± 4  105 ± 3  107 ± 4 |
| **pH 3.0** | 0.00  0.25  0.50  0.75  1.00 | 4.75 ± 0.22  5.12 ± 0.10  5.57 ± 0.21  6.07 ± 0.25  6.49 ± 0.18 | 44.0 ± 0.2  45.5 ± 0.2  47.1 ± 0.3  49.0 ± 0.4  50.8 ± 0.2 | 91 ± 4  92 ± 2  94 ± 4  96 ± 4  97 ± 3 |
| **pH 2.0** | 0.00  0.25  0.50  0.75  1.00 | 2.64 ± 0.13  2.99 ± 0.19  3.31 ± 0.15  3.61 ± 0.22  4.13 ± 0.19 | 36.0 ± 0.3  37.4 ± 0.4  38.8 ± 0.3  40.2 ± 0.2  42.5 ± 0.2 | 81 ± 4  82 ± 5  83 ± 4  84 ± 5  85 ± 4 |
|  | **[sorbitol]**  M | **Δ*C*_p_**  kcal mol^-1^ K^-1^ |  |  |
|  | 0.00  0.25  0.50  0.75  1.00 | 1.21 ± 0.04  1.18 ± 0.04  1.18 ± 0.08  1.18 ± 0.05  1.17 ± 0.06 |  |  |

*Values given in parentheses are from CD measurements.

**Table S4. Thermodynamic parameters associated with the thermal unfolding of RNase-A at different pH values in the presence and absence of taurine.**

| **pH** | **[taurine]**  M | **Δ*G*_D_^o^**  kcal mol^-1^ | ***T*_m_**  ^o^C | **Δ*H*_m_**  kcal mol^-1^ |
| --- | --- | --- | --- | --- |
| **pH 7.0** | 0.00  0.10  0.20  0.30  0.40 | 10.41 ± 0.14  10.48 ± 0.11  10.64 ± 0.32  10.83 ± 0.21  10.94 ± 0.18 | 63.0 ± 0.2  63.4 ± 0.4  63.7 ± 0.3  64.1 ± 0.1  64.5 ± 0.2  (64.0 ± 0.3)* | 116 ± 2  116 ± 2  117 ± 4  118 ± 3  118 ± 3  (120 ± 3) |
| **pH 6.0** | 0.00  0.10  0.20  0.30  0.40 | 10.10 ± 0.14  10.15 ± 0.23  10.30 ± 0.32  10.58 ± 0.21  10.67 ± 0.18 | 62.5 ± 0.3  62.8 ± 0.3  63.0 ± 0.4  63.3 ± 0.2  63.6 ± 0.3 | 114 ± 2  114 ± 3  115 ± 4  117 ± 3  117 ± 3 |
| **pH 5.0** | 0.00  0.10  0.20  0.30  0.40 | 9.31 ± 0.14  9.36 ± 0.22  9.53 ± 0.31  9.70 ± 0.20  9.79 ± 0.29 | 61.0 ± 0.2  61.3 ± 0.4  61.7 ± 0.2  62.1 ± 0.3  62.4 ± 0.2 | 109 ± 2  109 ± 3  110 ± 4  111 ± 3  111 ± 4 |
| **pH 4.0** | 0.00  0.10  0.20  0.30  0.40 | 7.47 ± 0.13  7.59 ± 0.21  7.63 ± 0.19  7.77 ± 0.19  7.95 ± 0.18 | 55.8 ± 0.4  56.0 ± 0.3  56.2 ± 0.3  56.5 ± 0.3  56.8 ± 0.2 | 99 ± 2  100 ± 3  100 ± 3  101 ± 3  102 ± 3 |
| **pH 3.0** | 0.00  0.10  0.20  0.30  0.40 | 4.75 ± 0.22  4.83 ± 0.21  4.97 ± 0.21  5.07 ± 0.15  5.30 ± 0.21 | 44.0 ± 0.2  44.4 ± 0.2  44.8 ± 0.3  45.3 ± 0.4  45.8 ± 0.2 | 91 ± 4  91 ± 4  92 ± 4  92 ± 3  94 ± 4 |
| **pH 2.0** | 0.00  0.10  0.20  0.30  0.40 | 2.64 ± 0.13  2.71 ± 0.14  2.85 ± 0.18  3.03 ± 0.18  3.14 ± 0.15 | 36.0 ± 0.3  36.3 ± 0.4  36.8 ± 0.3  37.3 ± 0.2  37.8 ± 0.2 | 81 ± 4  81 ± 4  82 ± 5  84 ± 5  84 ± 4 |
|  | **[taurine]**  M | **Δ*C*_p_**  kcal mol^-1^ K^-1^ |  |  |
|  | 0.00  0.10  0.20  0.30  0.40 | 1.21 ± 0.04  1.21 ± 0.05  1.21 ± 0.06  1.21 ± 0.06  1.19 ± 0.07 |  |  |

*Values given in parentheses are from CD measurements.

**Table S5.** Thermodynamic parameters associated with the thermal unfolding of lysozyme at different pH values in the presence and absence of urea.

| **pH** | **[urea]**  M | **∆*G*_D_^o^**  kcal mol^-1^ | | ***T*_m_**  ^o^C  (observed) (corrected) | | **∆*H*_m_**  kcal mol^-1^  (observed) (corrected) | |
| --- | --- | --- | --- | --- | --- | --- | --- |
| **pH 7.0** | 0.00  0.20  0.40  0.50  0.60  0.80  1.00  1.50  2.00 | 13.00 ± 0.23  12.87 ± 0.12  12.69 ± 0.34  12.67 ± 0.12  12.61 ± 0.29  12.43 ± 0.07  12.31 ± 0.18  12.06 ± 0.13  11.69 ± 0.08 | | 60.0 ± 0.2  59.3 ± 0.3  58.8 ± 0.2  58.4 ± 0.3  58.4 ± 0.3  57.9 ± 0.3  57.2 ± 0.3  56.4 ± 0.2  54.9 ± 0.3  (54.7 ± 0.2) | 86.0 ± 0.2  85.3 ± 0.3  84.8 ± 0.2  84.4 ± 0.3  84.4 ± 0.3  83.9 ± 0.2  83.2 ± 0.2  82.4 ± 0.2  80.9 ± 0.3  (80.7 ± 0.2)* | 93 ± 3  92 ± 2  91 ± 3  91 ± 2  90 ± 3  90 ± 3  89 ± 2  87 ± 2  85 ± 2  (85 ± 3) | 129 ± 3  128 ± 2  127 ± 3  127 ± 2  126 ± 3  125 ± 2  125 ± 2  123 ± 2  121 ± 2  (121 ± 3) |
| **pH 6.5** | 0.00  0.20  0.40  0.50  0.60  0.80  1.00  1.50  2.00 | 12.63 ± 0.24  12.51 ± 0.12  12.33 ± 0.18  12.32 ± 0.29  12.25 ± 0.13  12.07 ± 0.24  11.95 ± 0.18  11.68 ± 0.13  11.32 ± 0.24 | | 59.3 ± 0.2  58.7 ± 0.3  58.2 ± 0.2  57.9 ± 0.2  57.6 ± 0.4  57.1 ± 0.3  56.4 ± 0.2  55.2 ± 0.3  53.8 ± 0.2 | 84.9 ± 0.2  84.3 ± 0.3  83.8 ± 0.2  83.5 ± 0.2  83.2 ± 0.4  82.7 ± 0.3  82.0 ± 0.2  80.8 ± 0.3  79.4 ± 0.2 | 91 ± 3  90 ± 2  89 ± 2  89 ± 2  88 ± 2  87 ± 3  87 ± 2  85 ± 2  83 ± 3 | 127 ± 3  126 ± 2  125 ± 2  125 ± 3  124 ± 2  123 ± 3  123 ± 2  121 ± 2  119 ± 3 |
| **pH 6.0** | 0.00  0.20  0.40  0.50  0.60  0.80  1.00  1.50  2.00 | 12.43 ± 0.24  12.29 ± 0.13  12.11 ± 0.18  12.10 ± 0.29  12.01 ± 0.13  11.67 ± 0.24  11.55 ± 0.18  11.18 ± 0.13  10.74 ± 0.24 | | 58.5 ± 0.2  57.8 ± 0.3  57.2 ± 0.3  56.9 ± 0.2  56.5 ± 0.3  55.8 ± 0.3  55.0 ± 0.2  53.5 ± 0.3  51.7 ± 0.2 | 83.3 ± 0.2  82.6 ± 0.3  82.0 ± 0.3  81.7 ± 0.2  81.3 ± 0.3  80.6 ± 0.3  79.8 ± 0.2  78.3 ± 0.3  76.5 ± 0.2 | 90 ± 3  89 ± 2  88 ± 2  88 ± 2  87 ± 2  85 ± 3  85 ± 2  82 ± 2  80 ± 3 | 126 ± 3  125 ± 2  124 ± 2  124 ±3  123 ± 2  121 ± 3  121 ± 2  118 ± 2  116 ± 3 |
| **pH 5.5** | 0.00  0.20  0.40  0.50  0.60  0.80  1.00  1.50  2.00 | 12.04 ± 0.24  11.88 ± 0.29  11.73 ± 0.18  11.72 ± 0.28  11.48 ± 0.13  11.30 ± 0.24  11.19 ± 0.17  10.75 ± 0.28  10.10 ± 0.24 | | 57.0 ± 0.2  55.6 ± 0.3  55.8 ± 0.2  55.7 ± 0.2  55.2 ± 0.2  54.6 ± 0.2  53.9 ± 0.2  52.4 ± 0.3  51.0 ± 0.2 | 81.5 ± 0.2  80.1 ± 0.3  80.3 ± 0.2  80.2 ± 0.2  79.7 ± 0.2  79.1 ± 0.2  78.4 ± 0.2  76.9 ± 0.3  75.5 ± 0.2 | 91 ± 3  90 ± 3  89 ± 2  89 ± 3  87 ± 2  86 ± 3  86 ± 2  83 ± 3  79 ± 3 | 124 ± 3  123 ± 3  122 ± 2  122 ± 3  120 ± 2  119 ± 3  119 ± 2  116 ± 3  112 ± 3 |
| **pH 5.0** | 0.00  0.20  0.40  0.50  0.60  0.80  1.00  1.50  2.00 | 11.05 ± 0.24  10.91 ± 0.29  10.74 ± 0.33  10.72 ± 0.28  10.64 ± 0.13  10.31 ± 0.24  10.21 ± 0.17  9.78 ± 0.28  9.31 ± 0.24 | | 56.5 ± 0.2  55.6 ± 0.3  55.0 ± 0.3  54.5 ± 0.3  54.3 ± 0.2  53.6 ± 0.2  52.8 ± 0.1  51.1 ± 0.2  50.0 ± 0.3 | 80.0 ± 0.2  79.1 ± 0.2  78.5 ± 0.3  78.0 ± 0.2  77.8 ± 0.3  77.1 ± 0.3  76.3 ± 0.2  74.6 ± 0.3  73.5 ± 0.2 | 82 ± 3  81 ± 3  80 ± 3  80 ± 3  79 ± 2  77 ± 3  77 ± 3  74 ± 3  71 ± 3 | 118 ± 3  117 ± 3  116 ± 3  116 ± 3  115 ± 2  113 ± 3  113 ± 2  110 ± 3  107 ± 3 |
| **pH 4.0** | 0.00  0.20  0.40  0.50  0.60  0.80  1.00  1.50  2.00 | 10.30 ± 0.24  10.01 ± 0.27  9.84 ± 0.31  9.67 ± 0.27  9.74 ± 0.13  9.42 ± 0.37  9.19 ± 0.31  8.62 ± 0.14  8.16 ± 0.24 | | 52.5 ± 0.3  51.7 ± 0.3  51.0 ± 0.2  50.5 ± 0.2  50.3 ± 0.4  49.6 ± 0.3  49.0 ±0.3  47.1 ± 0.4  45.7 ± 0.4 | 75.9 ± 0.3  75.1 ± 0.3  74.4 ± 0.2  73.9 ± 0.2  73.7 ± 0.4  73.0 ± 0.3  72.4 ± 0.3  70.5 ± 0.4  69.1 ± 0.4 | 78 ± 3  76 ± 3  75 ± 3  74 ± 3  74 ± 2  72 ± 4  71 ± 4  67 ± 2  64 ± 3 | 114 ± 3  112 ± 3  111 ± 3  110 ± 3  110 ± 2  108 ± 4  107 ± 3  103 ± 2  100 ± 3 |
| **pH 3.0** | 0.00  0.20  0.40  0.50  0.60  0.80  1.00  1.50  2.00 | 7.86 ± 0.24  7.71 ± 0.27  7.54 ± 0.29  7.38 ± 0.26  7.30 ± 0.26  7.12 ± 0.35  6.90 ± 0.40  6.46 ± 0.25  5.83 ± 0.22 | | 70.3 ± 0.2  69.1 ± 0.3  68.0 ± 0.2  67.1 ± 0.2  66.9 ±0.3  65.8 ± 0.2  64.8 ± 0.4  62.1 ± 0.2  59.0 ± 0.2 | 70.3 ± 0.2  69.1 ± 0.3  68.0 ± 0.2  67.1 ± 0.2  66.9 ± 0.3  65.8 ± 0.2  64.8 ± 0.4  62.1 ± 0.2  59.0 ± 0.2 | 98 ± 3  97 ± 3  96 ± 3  95 ± 3  94 ± 3  93 ± 4  93 ± 4  89 ± 3  85 ± 3 | 98 ± 3  97 ± 3  96 ± 3  95 ± 3  94 ± 3  93 ± 4  93 ± 4  89 ± 3  85 ± 3 |
| **pH 2.0** | 0.00  0.20  0.40  0.50  0.60  0.80  1.00  1.50  2.00 | 5.69 ± 0.21  5.30 ± 0.23  5.01 ± 0.23  4.82 ± 0.12  4.75 ± 0.21  4.48 ± 0.27  4.18 ± 0.29  3.53 ± 0.18  3.02 ± 0.21 | | 57.7 ± 0.2  56.0 ± 0.4  54.6 ± 0.3  53.4 ± 0.2  53.3 ± 0.3  52.0 ± 0.2  50.6 ± 0.4  47.2 ± 0.2  44.5 ± 0.4 | 57.7 ± 0.2  56.0 ± 0.4  54.6 ± 0.3  53.4 ± 0.2  53.3 ± 0.3  52.0 ± 0.2  50.6 ± 0.4  47.2 ± 0.2  44.5 ± 0.4 | 85 ± 3  82 ± 2  80 ± 3  79 ± 2  78 ± 3  76 ± 4  74 ± 4  69 ± 3  65 ± 4 | 85 ± 3  82 ± 2  80 ± 3  79 ± 2  78 ± 3  76 ± 4  74 ± 4  69 ± 3  65 ± 4 |
|  | **[urea]**  M | **∆*C*_p_**  kcal mol^-1^ K^-1^ | |  |  |  |  |
|  | 0.00  0.20  0.40  0.50  0.60  0.80  1.00  1.50  2.00 | | 1.62 ± 0.05  1.61 ± 0.04  1.61 ± 0.03  1.61 ± 0.04  1.59 ± 0.04  1.59 ± 0.05  1.61 ± 0.03  1.59 ± 0.04  1.59 ± 0.05 |  | | | |

*Values given in parenthesis are from CD measurements.

**Table S6.** **Thermodynamic parameters associated with the thermal unfolding of lysozyme at different pH values in the presence and absence of myo-inositol.**

| **pH** | **[myo-inositol]**  M | **∆*G*_D_^o^**  kcal mol^-1^ | ***T*_m_**  ^o^C  (observed) (corrected) | | **∆*H*_m_**  kcal mol^-1^  (observed) (corrected) | |
| --- | --- | --- | --- | --- | --- | --- |
| **pH 7.0** | 0.00  0.10  0.20  0.30  0.40 | 13.00 ± 0.23  13.17 ± 0.06  13.31 ± 0.06  13.43 ± 0.18  13.56 ± 0.17 | 60.0 ± 0.2  60.4 ± 0.3  60.9 ± 0.2  61.3 ± 0.3  61.8 ± 0.3  (62.0 ± 0.2) | 86.0 ± 0.2  86.4 ± 0.3  86.9 ± 0.2  87.3 ± 0.3  87.8 ± 0.3  (88.0 ± 0.2)* | 93 ± 3  93 ± 2  94 ± 2  94 ± 3  95 ± 3  (94 ± 5) | 129 ± 3  129 ± 2  130 ± 2  130 ± 3  131 ± 3  (130 ± 5) |
| **pH 6.5** | 0.00  0.10  0.20  0.30  0.40 | 12.63 ± 0.23  12.81 ± 0.24  12.95 ± 0.23  13.07 ± 0.29  13.19 ± 0.29 | 59.3 ± 0.2  60.0 ± 0.3  60.6 ± 0.2  61.1 ± 0.4  61.5 ± 0.3 | 84.9 ± 0.2  85.6 ± 0.3  86.2 ± 0.2  86.7 ± 0.4  87.1 ± 0.3 | 91 ± 3  91 ± 3  92 ± 3  92 ± 4  93 ± 4 | 127 ± 3  127 ± 3  128 ± 3  128 ± 4  129 ± 4 |
| **pH 6.0** | 0.00  0.10  0.20  0.30  0.40 | 12.43 ± 0.24  12.61 ± 0.24  12.58 ± 0.24  12.86 ± 0.30  13.15 ± 0.46 | 58.5 ± 0.2  59.6 ± 0.5  60.2 ± 0.3  60.5 ± 0.4  60.7 ± 0.3 | 83.3 ± 0.2  84.4 ± 0.5  85.0 ± 0.3  85.3 ± 0.4  85.5 ± 0.3 | 90 ± 3  90 ± 3  90 ± 3  91 ± 4  93 ± 5 | 126 ± 3  126 ± 3  126 ± 3  127 ± 4  129 ± 5 |
| **pH 5.5** | 0.00  0.10  0.20  0.30  0.40 | 12.04 ± 0.24  12.23 ± 0.24  12.34 ± 0.40  12.63 ± 0.30  12.76 ± 0.30 | 57.0 ± 0.2  58.1 ± 0.3  58.4 ± 0.4  58.9 ± 0.3  59.2 ± 0.2 | 81.5 ± 0.2  82.6 ± 0.3  82.6 ± 0.4  83.4 ± 0.3  83.7 ± 0.2 | 88 ± 3  88 ± 3  89 ± 4  90 ± 4  91 ± 4 | 124 ± 3  124 ± 3  125 ± 4  126 ± 4  127 ± 4 |
| **pH 5.0** | 0.00  0.10  0.20  0.30  0.40 | 11.05 ± 0.24  11.36 ± 0.24  11.33 ± 0.24  11.60 ± 0.31  11.73 ± 0.30 | 56.5 ± 0.2  57.1 ± 0.4  57.6 ± 0.3  58.2 ± 0.2  58.8 ± 0.3 | 80.0 ± 0.2  80.6 ± 0.4  81.1 ± 0.3  81.7 ± 0.2  82.3 ± 0.3 | 82 ± 3  83 ± 3  83 ± 3  84 ± 4  85 ± 4 | 118 ± 3  119 ± 3  119 ± 3  120 ± 4  121 ± 4 |
|  | **[myo-inositol]**  M | **∆*C*_p_**  **kcal mol^-1^ K^-1^** |  |  |  |  |
|  | 0.00  0.10  0.20  0.30  0.40 | 1.62 ± 0.05  1.59 ± 0.03  1.60 ± 0.05  1.58 ± 0.07  1.59 ± 0.07 |  | | | |

*Values given in parenthesis are from CD measurements.

**Table S7.** **Thermodynamic parameters associated with the thermal unfolding of lysozyme at different pH values in the presence and absence of sorbitol.**

| **pH** | **[sorbitol]**  M | **∆*G*_D_^o^**  kcal mol^-1^ | | ***T*_m_**  ^o^C  (observed) (corrected) | | **∆*H*_m_**  kcal mol^-1^  (observed) (corrected) | |
| --- | --- | --- | --- | --- | --- | --- | --- |
| **pH 7.0** | 0.00  0.25  0.50  0.75  1.00 | 13.00 ± 0.23  13.30 ± 0.18  13.49 ± 0.23  13.80 ± 0.11  13.83 ± 0.11 | | 60.0 ± 0.2  60.7 ± 0.3  61.6 ± 0.4  62.5 ± 0.3  63.7 ± 0.2  (63.9 ± 0.2) | 86.0 ± 0.2  86.7 ± 0.3  87.6 ± 0.4  88.5 ± 0.3  89.7 ± 0.2 (89.9 ± 0.2)* | 93 ± 3  95 ± 4  95 ± 4  96 ± 3  96 ± 3  (95 ± 5) | 129 ± 3  131 ± 4  131 ± 4  132 ± 3  132 ± 3  (131 ± 5) |
| **pH 6.0** | 0.00  0.25  0.50  0.75  1.00 | 12.43 ± 0.24  12.90 ± 0.36  12.93 ± 0.24  13.24 ± 0.46  13.45 ± 0.46 | | 58.5 ± 0.2  59.7 ± 0.3  60.7 ± 0.4  61.8 ± 0.5  63.4 ± 0.3 | 83.3 ± 0.2  84.5 ± 0.3  85.5 ± 0.4  86.6 ± 0.5  88.2 ± 0.3 | 90 ± 3  92 ± 5  92 ± 4  93 ± 5  94 ± 5 | 126 ± 3  128 ± 5  128 ± 4  129 ± 5  130 ± 5 |
| **pH 5.0** | 0.00  0.25  0.50  0.75  1.00 | 11.05 ± 0.24  11.34 ± 0.37  11.53 ± 0.25  11.82 ± 0.30  11.86 ± 0.30 | | 56.5 ± 0.2  57.9 ± 0.3  59.2 ± 0.4  60.4 ± 0.2  62.2 ± 0.3 | 80.0 ± 0.2  81.4 ± 0.3  82.7 ± 0.4  83.9 ± 0.2  85.7 ± 0.3 | 82 ± 3  83 ± 5  84 ± 4  85 ± 4  85 ± 4 | 118 ± 3  119 ± 5  120 ± 4  121 ± 4  121 ± 4 |
| **pH 4.0** | 0.00  0.25  0.50  0.75  1.00 | 10.30 ± 0.24  10.58 ± 0.38  10.79 ± 0.26  11.07 ± 0.46  11.28 ± 0.30 | | 52.5 ± 0.3  53.9 ± 0.2  55.4 ± 0.3  56.6 ± 0.5  58.1 ± 0.4 | 75.9 ± 0.3  77.3 ± 0.2  78.8 ± 0.3  80.0 ± 0.5  81.5 ± 0.4 | 78 ± 3  79 ± 5  80 ± 4  81 ± 5  82 ± 4 | 114 ± 3  115 ± 5  116 ± 4  117 ± 5  118 ± 4 |
| **pH 3.0** | 0.00  0.25  0.50  0.75  1.00 | 7.86 ± 0.24  8.23 ± 0.38  8.42 ± 0.41  8.81 ± 0.41  8.85 ± 0.31 | | 70.3 ± 0.3  71.5 ± 0.2  73.0 ± 0.4  74.4 ± 0.3  75.8 ± 0.4 | 70.3 ± 0.2  71.5 ± 0.2  73.0 ± 0.4  74.4 ± 0.3  75.8 ± 0.4 | 98 ± 3  110 ± 5  101 ± 5  103 ± 5  103 ± 4 | 98 ± 3  100 ± 5  101 ± 5  103 ± 5  103 ± 4 |
| **pH 2.0** | 0.00  0.25  0.50  0.75  1.00 | 5.69 ± 0.21  5.99 ± 0.35  6.20 ± 0.37  6.54 ± 0.40  6.79 ± 0.41 | | 57.7 ± 0.3  58.5 ± 0.2  60.0 ± 0.4  61.4 ± 0.3  63.5 ± 0.4 | 57.7 ± 0.3  58.5 ± 0.2  60.0 ± 0.4  61.4 ± 0.3  63.5 ± 0.4 | 85 ± 3  87 ± 5  88 ± 5  90 ± 5  91 ± 5 | 85 ± 3  87 ± 5  88 ± 5  90 ± 5  91 ± 5 |
|  | **[sorbitol]**  M | **∆*C*_p_**  kcal mol^-1^ K^-1^ | |  |  |  |  |
|  | 0.00  0.25  0.50  0.75  1.00 | | 1.62 ± 0.05  1.60 ± 0.09  1.60 ± 0.08  1.58 ± 0.07  1.58 ± 0.07 |  | | | |

*Values given in parenthesis are from CD measurements.

**Table S8.** **Thermodynamic parameters associated with the thermal unfolding of lysozyme at different pH values in the presence and absence of taurine**.

| **pH** | **[taurine]**  M | **∆*G*_D_^o^**  kcal mol^-1^ | | ***T*_m_**  ^o^C  (observed) (corrected) | | **∆*H*_m_**  kcal mol^-1^  (observed) (corrected) | |
| --- | --- | --- | --- | --- | --- | --- | --- |
| **pH 7.0** | 0.00  0.10  0.20  0.30  0.40 | 13.00 ± 0.23  13.02 ± 0.13  13.14 ± 0.14  13.15 ± 0.24  13.33 ± 0.08 | | 60.0 ± 0.2  60.8 ± 0.2  61.3 ± 0.3  61.8 ± 0.2  62.3 ± 0.3  (62.5 ± 0.2) | 86.0 ± 0.2  86.8 ± 0.2  87.3 ± 0.3  87.8 ± 0.2  88.3 ± 0.3  (88.5 ± 0.2)* | 93 ± 3  93 ± 4  94 ± 4  94 ± 4  95 ± 3  (95 ± 5) | 129 ± 3  129 ± 4  130 ± 4  130 ± 4  131 ± 3  (131 ± 5) |
| **pH 6.0** | 0.00  0.10  0.20  0.30  0.40 | 12.43 ± 0.24  12.61 ± 0.31  12.74 ± 0.36  12.75 ± 0.24  12.93 ± 0.24 | | 58.5 ± 0.2  59.3 ± 0.3  60.0 ± 0.4  60.3 ± 0.3  60.8 ± 0.2 | 83.3 ± 0.2  84.1 ± 0.3  84.8 ± 0.4  85.1 ± 0.3  85.6 ± 0.2 | 90 ± 3  91 ± 5  92 ± 5  92 ± 4  93 ± 4 | 126 ± 3  127 ± 5  128 ± 5  128 ± 4  129 ± 4 |
| **pH 5.0** | 0.00  0.10  0.20  0.30  0.40 | 11.05 ± 0.24  11.23 ± 0.32  11.35 ± 0.21  11.53 ± 0.26  11.54 ± 0.25 | | 56.5 ± 0.2  57.3 ± 0.3  57.8 ± 0.5  58.4 ± 0.2  59.0 ± 0.3 | 80.0 ± 0.2  80.8 ± 0.3  81.3 ± 0.5  81.9 ± 0.2  82.5 ± 0.3 | 82 ± 3  83 ± 5  84 ± 4  85 ± 4  85 ± 4 | 118 ± 3  119 ± 5  120 ± 4  121 ± 4  121 ± 4 |
| **pH 4.0** | 0.00  0.10  0.20  0.30  0.40 | 10.30 ± 0.24  10.33 ± 0.19  10.46 ± 0.22  10.63 ± 0.27  10.64 ± 0.27 | | 52.5 ± 0.3  53.2 ± 0.2  53.8 ± 0.3  54.4 ± 0.5  54.9 ± 0.4 | 75.9 ± 0.2  76.6 ± 0.2  77.2 ± 0.3  77.8 ± 0.5  78.3 ± 0.4 | 78 ± 3  78 ± 4  79 ± 4  80 ± 4  80 ± 4 | 114 ± 3  114 ± 4  115 ± 4  116 ± 4  116 ± 4 |
| **pH 3.0** | 0.00  0.10  0.20  0.30  0.40 | 7.86 ± 0.24  7.88 ± 0.35  7.99 ± 0.24  8.17 ± 0.46  8.32 ± 0.27 | | 70.3 ± 0.3  71.0 ± 0.2  71.5 ± 0.4  72.7 ± 0.3  73.5 ± 0.4 | 70.3 ± 0.2  71.0 ± 0.2  71.5 ± 0.4  72.7 ± 0.3  73.5 ± 0.4 | 98 ± 3  98 ± 5  99 ± 4  100 ± 4  101 ± 4 | 98 ± 3  98 ± 5  99 ± 4  100 ± 4  101 ± 4 |
| **pH 2.0** | 0.00  0.10  0.20  0.30  0.40 | 5.69 ± 0.21  5.86 ± 0.33  5.99 ± 0.25  6.16 ± 0.16  6.19 ± 0.27 | | 57.7 ± 0.3  58.6 ± 0.2  59.4 ± 0.4  60.3 ± 0.3  60.9 ± 0.4 | 57.7 ± 0.3  58.6 ± 0.2  59.4 ± 0.4  60.3 ± 0.3  60.9 ± 0.4 | 85 ± 3  86 ± 5  87 ± 4  88 ± 3  88 ± 4 | 85 ± 3  86 ± 5  87 ± 4  88 ± 3  88 ± 4 |
|  | **[taurine]**  M | **∆*C*_p_**  kcal mol^-1^ K^-1^ | |  |  |  |  |
|  | 0.00  0.10  0.20  0.30  0.40 | | 1.62 ± 0.05  1.62 ± 0.10  1.63 ± 0.09  1.63 ± 0.08  1.63 ± 0.08 |  | | | |

*Values given in parenthesis are from CD measurements.

**Table S9. Thermodynamic parameters associated with the thermal unfolding of α-LA at different pH values in the presence and absence of urea.**

| **pH** | **[urea]**  M | **Δ*G*_D_^o^**  kcal mol^-1^ | ***T*_m_**  ^o^C | **Δ*H*_m_**  kcal mol^-1^ |
| --- | --- | --- | --- | --- |
| **pH 7.0** | 0.00  0.20  0.40  0.50  0.60  0.80 | 2.01 ± 0.12  1.95 ± 0.16  1.80 ± 0.10  1.54 ± 0.16  1.71 ± 0.10  1.60 ± 0.12 | 42.3 ± 0.3  41.3 ± 0.2  40.6 ± 0.5  40.1 ± 0.2  39.9 ± 0.3  39.0 ± 0.2 | 50 ± 3  49 ± 4  47 ± 3  44 ± 4  46 ± 3  45 ± 3 |
| **pH 6.5** | 1.00  1.50  2.00  0.00  0.20  0.40  0.50  0.60  0.80  1.00  1.50  2.00 | 1.47 ± 0.10  1.21 ± 0.10  0.91 ± 0.12  3.71 ± 0.15  3.69 ± 0.21  3.47 ± 0.11  3.34 ± 0.21  3.35 ± 0.19  3.13 ± 0.14  2.96 ± 0.19  2.57 ± 0.16  2.10 ±0.18 | 38.0 ± 0.5  36.2 ± 0.3  35.0 ± 0.2  (34.8 ± 0.2)*  50.5 ± 0.2  49.4 ± 0.5  48.5 ± 0.2  48.1 ± 0.3  47.6 ± 0.2  46.4 ± 0.3  46.2 ± 0.2  44.2 ± 0.2  41.5 ± 0.3 | 42 ± 3  39 ± 3  35 ± 3  (34 ± 3)  67 ± 3  66 ± 4  64 ± 3  65 ± 4  63 ± 4  61 ± 3  58 ± 4  52 ± 3  49 ± 4 |
| **pH 6.0** | 0.00  0.20  0.40  0.50  0.60  0.80  1.00  1.50  2.00 | 4.25 ± 0.16  4.20 ± 0.22  3.98 ± 0.11  3.91 ± 0.06  3.80 ± 0.12  3.68 ± 0.16  3.51 ± 0.12  3.12 ± 0.11  2.71 ± 0.16 | 55.0 ± 0.2  54.0 ± 0.4  53.2 ± 0.2  52.9 ± 0.2  52.3 ± 0.3  51.3 ± 0.3  51.1 ± 0.3  49.6 ± 0.4  47.7 ± 0.3 | 70 ± 3  70 ± 4  68 ± 3  65 ± 2  66 ± 3  64 ± 3  62 ± 3  58 ± 2  54 ± 3 |
| **pH 5.5** | 0.00  0.20  0.40  0.50  0.60  0.80  1.00  1.50  2.00 | 5.03 ± 0.16  5.09 ± 0.23  4.86 ± 0.19  4.77 ± 0.24  4.65 ± 0.11  4.67 ± 0.16  4.39 ± 0.12  4.00 ± 0.13  3.62 ± 0.18 | 59.8 ± 0.4  59.0 ± 0.2  58.3 ± 0.3  57.0 ± 0.3  57.0 ± 0.3  56.6 ± 0.4  55.9 ± 0.3  55.6 ± 0.3  53.3 ± 0.2 | 77 ± 3  74 ± 4  72 ± 4  71 ± 4  70 ± 3  70 ± 3  68 ± 3  64 ± 2  60 ± 3 |
|  | **[urea]**  M | **Δ*C*_p_**  kcal mol^-1^ K^-1^ |  |  |
|  | 0.00  0.20  0.40  0.50  0.60  0.80  1.00  1.50  2.00 | 1.51 ± 0.08  1.38 ± 0.10  1.37 ± 0.12  1.37 ± 0.09  1.33 ± 0.11  1.30 ± 0.08  1.35 ± 0.11  1.30 ± 0.04  1.27 ± 0.06 |  |  |

*Values given in parenthesis are from CD measurements.

**Table S 10.** **Thermodynamic parameters associated with the thermal unfolding of α-LA at different pH values in the presence and absence of myo-inositol.**

| **pH** | **[myo-inositol]**  M | **Δ*G*_D_^o^**  kcal mol^-1^ | ***T*_m_**  ^o^C | **Δ*H*_m_**  kcal mol^-1^ |
| --- | --- | --- | --- | --- |
| **pH 7.0** | 0.00  0.10  0.20  0.30  0.40 | 2.01 ± 0.13  2.12 ± 0.17  2.21 ± 0.18  2.36 ± 0.13  2.43 ± 0.14 | 42.3 ± 0.2  43.2 ± 0.2  43.8 ± 0.2  44.6 ± 0.4  45.3 ± 0.3  (45.5 ± 0.4)* | 50 ± 3  51 ± 4  52 ± 4  53 ± 3  54 ± 3  (55 ± 3) |
| **pH 6.5** | 0.00  0.10  0.20  0.30  0.40 | 3.71 ± 0.15  3.83 ± 0.19  4.05 ± 0.21  4.27 ± 0.15  4.26 ± 0.25 | 50.5 ± 0.2  51.2 ± 0.4  52.1 ± 0.4  53.1 ± 0.2  53.9 ± 0.3 | 67 ± 3  68 ± 4  70 ± 4  71 ± 3  71 ± 4 |
| **pH 6.0** | 0.00  0.10  0.20  0.30  0.40 | 4.25 ± 0.16  4.36 ± 0.20  4.60 ± 0.12  4.71 ± 0.15  4.88 ± 0.16 | 55.0 ± 0.2  55.7 ± 0.3  56.5 ± 0.2  57.3 ± 0.3  58.1 ± 0.2 | 70 ± 3  71 ± 4  73 ± 3  73 ± 3  75 ± 3 |
| **pH 5.5** | 0.00  0.10  0.20  0.30  0.40 | 5.03 ± 0.16  5.20 ± 0.19  5.32 ± 0.21  5.59 ± 0.14  5.62 ± 0.16 | 59.8 ± 0.2  60.5 ± 0.3  61.3 ± 0.3  62.1 ± 0.2  62.8 ± 0.3 | 76 ± 3  77 ± 4  78 ± 4  79 ± 3  80 ± 3 |
|  | **[myo-inositol]**  M | **Δ*C*_p_**  kcal mol^-1^ K^-1^ |  |  |
|  | 0.00  0.10  0.20  0.30  0.40 | 1.51 ± 0.08  1.53 ± 0.12  1.54 ± 0.11  1.48 ± 0.09  1.53 ± 0.08 |  |  |

*Values given in parentheses are from CD measurements.

**Table S 11.** **Thermodynamic parameters associated with the thermal unfolding of α-LA at different pH values in the presence and absence of sorbitol.**

| **pH** | **[sorbitol]**  M | **Δ*G*_D_^o^**  kcal mol^-1^ | ***T*_m_**  ^o^C | **Δ*H*_m_**  kcal mol^-1^ |
| --- | --- | --- | --- | --- |
| **pH 7.0** | 0.00  0.25  0.50  0.75  1.00 | 2.01 ± 0.13  2.24 ± 0.17  2.55 ± 0.12  2.79 ± 0.06  3.07 ± 0.14 | 42.3 ± 0.2  44.1 ± 0.2  45.9 ± 0.3  47.7 ± 0.4  50.1 ± 0.4  (50.1 ± 0.3)* | 50 ± 3  52 ± 4  55 ± 3  57 ± 2  59 ± 3  (58 ± 3) |
| **pH 6.5** | 0.00  0.25  0.50  0.75  1.00 | 3.71 ± 0.16  4.10 ± 0.11  4.49 ± 0.12  4.78 ± 0.23  5.28 ± 0.25 | 50.5 ± 0.2  52.4 ± 0.4  54.2 ± 0.4  55.9 ± 0.2  57.8 ± 0.3 | 67 ± 3  70 ± 3  72 ± 3  74 ± 4  76 ± 4 |
| **pH 6.0** | 0.00  0.25  0.50  0.75  1.00 | 4.25 ± 0.16  4.51 ± 0.20  4.83 ± 0.11  5.40 ± 0.23  5.71 ± 0.14 | 55.0 ± 0.2  56.9 ± 0.3  58.8 ± 0.2  61.7 ± 0.2  63.0 ± 0.2 | 70 ± 3  72 ± 4  74 ± 3  77 ± 4  79 ± 3 |
| **pH 5.5** | 0.00  0.25  0.50  0.75  1.00 | 5.03 ± 0.16  5.32 ± 0.19  5.48 ± 0.21  5.64 ± 0.23  5.91 ± 0.25 | 59.8 ± 0.2  61.7 ± 0.3  63.5 ± 0.3  65.3 ± 0.3  67.2 ± 0.5 | 76 ± 3  80 ± 4  82 ± 4  84 ± 4  86 ± 4 |
|  | **[sorbitol]**  M | **Δ*C*_p_**  kcal mol^-1^ K^-1^ |  |  |
|  | 0.00  0.25  0.50  0.75  1.00 | 1.51 ± 0.08  1.54 ± 0.12  1.53 ± 0.11  1.48 ± 0.10  1.48 ± 0.09 |  |  |

*Values given in parentheses are from CD measurements.

**Table S 12.** **Thermodynamic parameters associated with the thermal unfolding of α-LA at different pH values in the presence and absence of taurine.**

| **pH** | **[taurine]**  M | **Δ*G*_D_^o^**  kcal mol^-1^ | ***T*_m_**  ^o^C | **Δ*H*_m_**  kcal mol^-1^ |
| --- | --- | --- | --- | --- |
| **pH 7.0** | 0.00  0.10  0.20  0.30  0.40 | 2.01 ± 0.13  2.09 ± 0.17  2.19 ± 0.12  2.29 ± 0.12  2.40 ± 0.20 | 42.3 ± 0.2  42.8 ± 0.2  44.0 ± 0.3  44.4 ± 0.2  45.4 ± 0.3  (45.1 ± 0.3)* | 50 ± 3  51 ± 4  51 ± 3  52 ± 3  53 ± 4  (53 ± 3) |
| **pH 6.5** | 0.00  0.10  0.20  0.30  0.40 | 3.71 ± 0.15  3.86 ± 0.20  4.02 ± 0.21  4.19 ± 0.13  4.36 ± 0.24 | 50.5 ± 0.2  51.2 ± 0.4  52.4 ± 0.4  53.1 ± 0.3  54.0 ± 0.3 | 67 ± 3  68 ± 4  68 ± 4  70 ± 3  72 ± 4 |
| **pH 6.0** | 0.00  0.10  0.20  0.30  0.40 | 4.25 ± 0.16  4.51 ± 0.12  4.69 ± 0.21  4.68 ± 0.23  4.96 ± 0.25 | 55.0 ± 0.2  55.9 ± 0.3  57.4 ± 0.2  58.5 ± 0.2  59.9 ± 0.2 | 70 ± 3  72 ± 3  73 ± 4  73 ± 4  75 ± 4 |
| **pH 5.5** | 0.00  0.10  0.20  0.30  0.40 | 5.03 ± 0.16  5.25 ± 0.21  5.51 ± 0.21  5.45 ± 0.12  5.73 ± 0.14 | 59.8 ± 0.2  60.4 ± 0.3  61.3 ± 0.3  62.0 ± 0.2  62.7 ± 0.2 | 76 ± 3  77 ± 3  79 ± 3  79 ± 3  81 ± 3 |
|  | **[taurine]**  M | **Δ*C*_p_**  kcal mol^-1^ K^-1^ |  |  |
|  | 0.00  0.10  0.20  0.30  0.40 | 1.51 ± 0.08  1.50 ± 0.11  1.50 ± 0.11  1.54 ± 0.10  1.53 ± 0.09 |  |  |

*Values given in parentheses are from CD measurements.

**Table S 13.Thermal denaturation studies on proteins to see the counteracting effect of sorbitol with increasing urea concentration at pH 7.0^a^**

| [sorbitol]  M | [urea]  M | *T*_m_  ^o^C | | |
| --- | --- | --- | --- | --- |
|  |  | RNase-A | lysozyme | α-LA |
| 0.00 | 0.00 | 63.0 ± 0.3 | 86.0 ± 0.3 | 42.3 ± 0.2 |
|  |  |  |  |  |
| 0.00  0.25  0.50  1.00  2.00  2.25  2.50  3.00  3.50  3.75 | 0.30  0.30  0.30  0.30  0.30  0.30  0.30  0.30  0.30  0.30 | 62.2 ± 0.3  62.0 ± 0.3  62.0 ± 0.4  62.2 ± 0.3  -  -  63.5 ± 0.3  -  64.3 ± 0.3  - | 85.4 ± 0.3  85.2 ± 0.3  85.0 ± 0.4  85.3 ± 0.3  85.5 ± 0.3  85.8 ± 0.3  -  -  -  - | 40.8 ± 0.4  40.8 ± 0.3  40.7 ± 0.2  40.8 ± 0.3  41.5 ± 0.3  -  42.7 ± 0.4  43.4 ± 0.3  -  44.0 ± 0.2 |
|  |  |  |  |  |
| 0.00  0.25  0.50  1.00  2.00  2.25  2.50  3.00  3.50  3.75 | 0.40  0.40  0.40  0.40  0.40  0.40  0.40  0.40  0.40  0.40 | 61.8 ± 0.2  -  61.8 ± 0.3  61.9 ± 0.3  -  -  62.5 ± 0.4  -  63.1 ± 0.4  - | 84.8 ± 0.2  -  84.6 ± 0.3  84.8 ± 0.3  84.5 ± 0.4  84.8 ± 0.4  -  -  -  - | 40.5 ± 0.2  40.5 ± 0.3  40.3 ± 0.3  40.4 ± 0.4  40.5 ± 0.2  -  40.9 ± 0.4  42.0 ± 0.3  -  42.7 ± 0.4 |
| 0.00  0.25  0.50  0.75  1.00  2.00  2.25  2.50  3.00  3.50  3.75 | 0.50  0.50  0.50  0.50  0.50  0.50  0.50  0.50  0.50  0.50  0.50 | 61.6 ± 0.3  -  61.5 ± 0.3  -  61.3 ± 0.4  -  -  61.5 ± 0.3  -  62.1 ± 0.3  - | 84.4 ± 0.2  -  84.0 ± 0.3  -  84.3 ± 0.4  84.6 ± 0.3  84.7 ± 0.3  -  -  -  - | 40.1 ± 0.2  39.8 ± 0.3  39.8 ± 0.4  39.6 ± 0.3  39.8 ± 0.3  40.0 ± 0.2  -  40.5 ± 0.3  40.8 ± 0.4  -  41.6 ± 0.5 |
| 0.00  1.00  2.00  2.50  3.00  3.25  3.50 | 1.00  1.00  1.00  1.00  1.00  1.00  1.00 | 60.0 ± 0.4  -  -  59.8 ± 0.2  -  60.2 ± 0.3  - | 83.2 ± 0.3  82.8 ± 0.2  83.0 ± 0.3  -  -  -  - | 38.0 ± 0.3  -  37.8 ± 0.2  -  37.5 ± 0.3  -  37.6 ± 0.2 |

^a^A ± with *T*_m_ is the mean error obtained from the triplicate measurements.

**FIGURE S1.**

**FIGURE S2.**

**FIGURE S3.**

**FIGURE S4.**

**FIGURE S5.**

**FIGURE S6.**

**FIGURE S7.**

**FIGURE LEGENDS**

**FIGURE S1. Effect of urea, osmolyte and their mixture on RNase-A**. Thermal denaturation curves of RNase-A, in the presence of the indicated concentrations of urea, osmolyte, and their mixture at the predicted molar ratio of urea: osmolyte (indicated in the figure), monitored by change in mean residue ellipticity at 222 nm at pH 7.0. To maintain clarity all data point are not shown.

**FIGURE S2. Effect of urea, osmolyte and their mixture on Lysozyme.** Thermal denaturation curves of lysozyme, in the presence of the indicated concentrations of urea, osmolyte, and their mixture at the predicted molar ratio of urea: osmolyte (indicated in the figure), monitored by change in mean residue ellipticity at 222 nm at pH 7.0. To maintain clarity all data point are not shown.

**FIGURE S3. Effect of urea, osmolyte and their mixture on α-LA**. Thermal denaturation curves of α-LA, in the presence of the indicated concentrations of urea, osmolyte, and their mixture at the predicted molar ratio of urea: osmolyte (indicated in the figure), monitored by change in mean residue ellipticity at 222 nm at pH 7.0. To maintain clarity all data point are not shown.

**FIGURE S4. Effect of urea, myo-inositol and their mixture on secondary and tertiary structures of proteins at pH 7.0**. Panels A and D, B and E, and C and F represent data of RNase-A, lysozyme and α-LA, respectively. Curve 1 depicts the far- and near-UV CD spectra of the native state in 0 M myo-inositol (—), 0.40 M myo-inositol (—. —), 0.80 M urea (**….**) and molar ratio of myo-inositol: urea (see Table 2) (—..—) at 25 ^o^C. Curve 2 depicts the far- and near-UV CD spectra of the denatured state of proteins at 85 ^o^C, and symbols have the same meaning as in Curve 1.

**FIGURE S5. Effect of urea, sorbitol and their mixture on secondary and tertiary structures of proteins at pH 7.0.** Panels A and D, B and E, and C and F represent data of RNase-A, lysozyme and α-LA, respectively. Curve 1 depicts the far- and near-UV CD spectra of the native state in 0 M sorbitol (—), 1.00 M sorbitol (—. —), 2.00 M urea (**….**) and molar ratio of sorbitol: urea (see Table 2) (—..—) at 25 ^o^C. Curve 2 depicts the far- and near-UV CD spectra of the denatured state of proteins at 85 ^o^C, and symbols have the same meaning as in Curve 1..

**FIGURE S6. Effect of urea, taurine and their mixture on secondary and tertiary structures of proteins at pH 7.0.** Panels A and D, B and E, and C and F represent data of RNase-A, lysozyme and α-LA, respectively. Curve 1 depicts the far- and near-UV CD spectra of the native state in 0 M taurine (—), 0.40 M taurine (—. —), 0.80 M urea (**….**) and molar ratio of taurine: urea (see Table 2) (—..—) at 25 ^o^C. Curve 2 depicts the far- and near-UV CD spectra of the denatured state of proteins at 85 ^o^C, and symbols have the same meaning as in Curve 1.

**FIGURE S7. Effect of urea, sorbitol and their mixtures (at different ratios) on proteins at pH 7.0**. Representative thermal denaturation curves of RNase-A, lysozyme and α-LA in the presence of the indicated concentrations of urea, sorbitol, and their mixtures. To maintain clarity all data points and transition curves are not shown.
